# Exponential Stability and Hypoelliptic Regularization for the Kinetic Fokker–Planck Equation with Confining Potential

**DOI:** 10.1007/s10955-024-03263-2

**Published:** 2024-04-27

**Authors:** Anton Arnold, Gayrat Toshpulatov

**Affiliations:** Institute for Analysis and Scientific Computing, TU Wien, Wiedner Hauptstraße 8-10, 1040 Vienna, Austria

**Keywords:** Kinetic theory, Fokker–Planck equation, Confinement potential, Degenerate evolution, Long time behavior, Convergence to equilibrium, Hypocoercivity, Hypoelliptic regularity, Lyapunov functional, 35Q84, 35B40, 35Q82, 82C40

## Abstract

This paper is concerned with a modified entropy method to establish the large-time convergence towards the (unique) steady state, for kinetic Fokker–Planck equations with non-quadratic confinement potentials in whole space. We extend previous approaches by analyzing Lyapunov functionals with non-constant weight matrices in the dissipation functional (a generalized Fisher information). We establish exponential convergence in a weighted $H^1$-norm with rates that become sharp in the case of quadratic potentials. In the defective case for quadratic potentials, i.e. when the drift matrix has non-trivial Jordan blocks, the weighted $L^2$-distance between a Fokker–Planck-solution and the steady state has always a sharp decay estimate of the order $\mathcal O\big ( (1+t)e^{-t\nu /2}\big )$, with $\nu$ the friction parameter. The presented method also gives new hypoelliptic regularization results for kinetic Fokker–Planck equations (from a weighted $L^2$-space to a weighted $H^1$-space).

## Introduction

This paper is devoted to the study of the long time behavior of the kinetic Fokker–Planck equation

1 $$\begin{aligned} {\left\{ \begin{array}{ll} \partial _t f+v \cdot \nabla _x f-\nabla _x V \cdot \nabla _v f=\nu \text {div}_v(vf)+\sigma \Delta _v f,\, \, \, x,\,v \in \mathbb {R}^n,\, t>0\\ f(t=0)=f_0 \in L^1(\mathbb {R}^{2n}) \end{array}\right. } \end{aligned}$$

describing the time evolution of the phase space probability density *f*(*t*, *x*, *v*),  e.g. in a plasma [30]. Applications range from plasma physics [13, 29] to stellar dynamics [17, 18]. Here $V=V(x)$ is a given smooth, bounded below confinement potential for the system, and $\nu>0, \,\sigma >0$ denote the friction and diffusion parameters, respectively. This equation is associated with the Langevin stochastic differential equation

$$\begin{aligned} {\left\{ \begin{array}{ll} dx_t=v_tdt\\ dv_t=-\nu v_t dt-\nabla V(x_t)dt+\sqrt{2\sigma } dB_t, \end{array}\right. } \end{aligned}$$

where $\{B_t\}_{t\ge 0}$ is a Brownian motion in $\mathbb {R}^n$ with covariance $\langle B_t,B_{t'}\rangle =\delta _{t-t'}.$

Since the equation conserves mass, i.e.,

$$\begin{aligned} \int _{\mathbb {R}^{2n}}f(t,x,v) dxdv=\int _{\mathbb {R}^{2n}}f_0(x,v) dxdv,\, \, \, \, \, t\ge 0, \end{aligned}$$

we shall always assume (without restriction of generality) that $\displaystyle \int _{\mathbb {R}^{2n}}f_0(x,v) dxdv=1.$ The unique normalized steady state of (1) is given by

2 $$\begin{aligned} f_{\infty }(x,v)=c_Ve^{-\frac{\nu }{\sigma }[V(x)+\frac{|v|^2}{2}]},\, \, \, x,v \in \mathbb {R}^n, \end{aligned}$$

where $c_V$ is a positive constant such that $\int _{\mathbb {R}^{2n}} f_{\infty }(x,v)dxdv=1.$ The following equation is also considered as the kinetic Fokker–Planck equation:

3 $$\begin{aligned} \partial _t h+v \cdot \nabla _x h-\nabla _x V \cdot \nabla _v h=\sigma \Delta _v h-\nu v\cdot \nabla _v h,\, \, \, x,\,v \in \mathbb {R}^n,\, t>0, \end{aligned}$$

and to switch from (1) to (3) it suffices to set $h:=f/f_{\infty }.$

It was shown in [22] that, if $V\in C^{\infty }(\mathbb {R}^n),$ (3) generates a $C^{\infty }$ regularizing contraction semigroup in $L^2(\mathbb {R}^d, f_{\infty }):=\{g:\mathbb {R}^d \rightarrow \mathbb {R}: g \text { is measurable and } \int _{\mathbb {R}^d}g^2f_{\infty }dxdv< \infty \}, \, \, \, d=2n.$ For well-posedness with non-smooth potentials, we refer to [31, Theorems 6, 7].

The long time behavior and exponential convergence of the solution to the steady state has been studied and there are various results: in [19], algebraic decay was proved for potentials that are asymptotically quadratic (as $|x|\rightarrow \infty$) and for initial conditions that are bounded below and above by Gaussians. The authors used logarithmic Sobolev inequalities and entropy methods. In [24], exponential decay was obtained also for faster growing potentials and more general initial conditions. That proof is based on hypoellipticity techniques. By using hypoelliptic methods, Villani proved exponential convergence results in $H^1(\mathbb {R}^d, f_{\infty }):=\{g \in L^2(\mathbb {R}^d, f_{\infty }): |\nabla g| \in L^2(\mathbb {R}^d, f_{\infty })\}$ [31, Theorem 35] and in $L^2(\mathbb {R}^d, f_{\infty })$ [31, Theorem 37]. The main conditions in Villani’s theorems above, as well as in [9, 10, 14, 15, 20, 32], are the validity of the Poincaré inequality (5) and the criterion

4 $$\begin{aligned} \exists \, \, C\ge 0\, \,: \, \, \, \, \, \, \, \, \left| \left| \frac{\partial ^2 V(x)}{\partial x^2}\right| \right| \le C(1+|\nabla V(x)|),\, \, \, \, \forall x \in \mathbb {R}^n, \end{aligned}$$

where $\displaystyle \left| \left| \frac{\partial ^2 V(x)}{\partial x^2}\right| \right|$ denotes the Frobenius norm of $\displaystyle \frac{\partial ^2 V(x)}{\partial x^2}.$

When $\frac{\partial ^2 V}{\partial x^2}$ is bounded, Villani also proved that the solution converges to the steady state exponentially in the logarithmic entropy [31, Theorem 39]. This result was extended in [16] to potentials *V* satisfying a weighted log-Sobolev inequality and the condition that $V^{-2\eta }\frac{\partial ^2 V}{\partial x^2}$ is bounded for some $\eta \ge 0.$ Even though Villani’s result allows for a general class of potentials, the growth condition (4) is not satisfied by potentials with singularities. This type of potentials, such as Lennard–Jones type interactions with confinement, are considered in [10] and their method relies on an explicit construction of a Lyapunov function and Gamma calculus. In [20], Dolbeault et al. developed a method to get exponential decay in $L^2$ for a large class of linear kinetic equations, including (1). Their method was also used to study the long time behavior of (1) when the potential *V* is zero or grows slowly as $|x|\rightarrow \infty ,$ see [11, 12]. Based on a probabilistic coupling method, Eberle et al. [21] obtained an exponential decay result in Wasserstein distance.

The associated semigroup of the kinetic Fokker–Planck equation has instantaneous regularizing properties which is called *hypoellipticity* [26]. This hypoelliptic regularization is obvious when the confining potential *V* is zero or quadratic as the fundamental solution can be explicitly computed (see [26, 28]). For potentials such that $\frac{\partial ^2 V}{\partial x^2}$ is bounded, Hérau [23] obtained short time estimates for a $L^2(\mathbb {R}^d, f_{\infty })\rightarrow H^1(\mathbb {R}^d, f_{\infty })$ regularization by constructing a suitable Lyapunov functional. Based on interpolation inequalities and a system of differential inequalities, Villani [31, Appendix A.21] extended Hérau’s result for potentials satisfying (4).

We provide a new method to establish exponential decay of the solution to the steady state in $H^1(\mathbb {R}^d, f_{\infty })$ for a wide class of potentials: Our method extends [1, 3, 31] by allowing for more general Lyapunov functionals. Generalizing the previous approaches, the weight matrix in the dissipation functional (a generalized Fisher information) may now depend on *x* and *v*. This leads to a new criterion on the potential *V*. For this entropy method we need the time derivative of the dissipation functional, but we also provide its (*x*, *v*)–pointwise analog, in the spirit of the *Gamma calculus* [9]. We provide a formula to estimate easily the exponential decay rate depending on the parameters of the equation, the constants appearing in the Poincaré inequality (5) and the growth condition on the potential (see (6) below). As a test of the effectiveness of our method, we show that our estimate on the decay rate is sharp when the potential is a quadratic polynomial. Moreover, our method lets us obtain estimates on the hypoelliptic regularization for potentials that are more general than in [23].

The organization of this paper is as follows. In Sect. 2, we define the assumptions on the potential, state the main results, and present concrete examples of such potentials. In Sect. 3, we present the intuition and explain our method. Section 4 contains important lemmas about matrix inequalities which are important to construct suitable Lyapunov functionals. The final section presents the proof of the main results.

## Main Results

We make the following assumptions.

### Assumption 2.1

There exists a constant $C_{PI}>0$ such that the Poincaré inequality

5 $$\begin{aligned} \int _{\mathbb {R}^{2n}} h^2 f_{\infty }dxdv-\left( \int _{\mathbb {R}^{2n}} h f_{\infty }dxdv\right) ^2\le \frac{1}{C_{PI}}\int _{\mathbb {R}^{2n}} \big (|\nabla _x h|^2+|\nabla _v h|^2\big )f_{\infty }dxdv\, \, \end{aligned}$$

holds for all $h \in H^1(\mathbb {R}^d, f_{\infty }).$

Sufficient conditions on the potential appearing in $f_{\infty }$ so that the Poincaré inequality holds, e.g. the Bakry–Emery criterion, are presented in [8, Chapter 4].

### Assumption 2.2

There are constants $$c\in \mathbb {R}$$ and $$\tau \in [0,\nu ) $$ such that the following $$\mathbb {R}^{m\times m}$$ matrix, $$ m{:}{=}n(n+1),$$

6 $$\begin{aligned} \begin{pmatrix} \nu \left( \frac{\partial ^2 V(x)}{\partial x^2}+cI\right) &{}0&{}...&{}0&{}-\frac{1}{2}\frac{\partial ^2(\partial _{x_1}V(x))}{\partial x^2} \\ 0&{}\nu \left( \frac{\partial ^2 V(x)}{\partial x^2}+cI\right) &{}...&{}0&{}-\frac{1}{2}\frac{\partial ^2(\partial _{x_2}V(x))}{\partial x^2}\\ ...&{}...&{}...&{}...&{}...\\ 0&{}0&{}...&{}\nu \left( \frac{\partial ^2 V(x)}{\partial x^2}+cI\right) &{}-\frac{1}{2}\frac{\partial ^2(\partial _{x_n}V(x))}{\partial x^2}\\ -\frac{1}{2}\frac{\partial ^2(\partial _{x_1}V(x))}{\partial x^2}&{}-\frac{1}{2}\frac{\partial ^2(\partial _{x_2}V(x))}{\partial x^2}&{}...&{}-\frac{1}{2}\frac{\partial ^2(\partial _{x_n}V(x))}{\partial x^2}&{}\frac{\tau \nu }{2\sigma }\left( \frac{\partial ^2 V(x)}{\partial x^2}+cI\right) \end{pmatrix} \end{aligned}$$

is positive semi-definite for all $x \in \mathbb {R}^n,$ where $I\in \mathbb {R}^{n \times n}$ denotes the identity matrix.

Roughly speaking, Assumption 2.2 essentially means that the second order derivatives of *V* control the third order ones. It implies that $\frac{\partial ^2 V(x)}{\partial x^2}+ cI$ is positive semi-definite for all $x \in \mathbb {R}^n,$ and hence the eigenvalues of $\frac{\partial ^2 V(x)}{\partial x^2}$ are uniformly bounded from below. We note that, in contrast to the Bakry–Emery strategy [7], the eigenvalues here may take negative values.

Let $\displaystyle \alpha (x)\in \mathbb {R}$ denote the smallest eigenvalue of $\frac{\partial ^2 V(x)}{\partial x^2}$ at $x \in \mathbb {R}^n.$ Then the following condition implies Assumption 2.2. For its proof see Appendix 6.1.

**Assumption 2.2’** *There are constants* $c\in \mathbb {R}$ and $\tau \in [ 0,\nu )$ *such that* $\frac{\partial ^2 V(x)}{\partial x^2}+cI$ *is positive semi-definite and*^1^

7 $$\begin{aligned} -\sqrt{\frac{2\tau \nu ^2 }{n\sigma }}(\alpha (x)+ c)I\le \frac{\partial ^2 (\partial _{x_i}V(x))}{\partial x^2} \le \sqrt{\frac{2\tau \nu ^2}{n \sigma }}(\alpha (x)+ c)I \end{aligned}$$

* for all* $x \in \mathbb {R}^n$ *and* $i\in \{1,...,n\}.$

We denote

8 $$\begin{aligned} \displaystyle \alpha _0{:}{=}\inf _{x \in \mathbb {R}^n}\alpha (x) \end{aligned}$$

and assume in the sequel that $\alpha _0>- \infty .$ Hence Assumption 2.2 can only hold for some $c\ge -\alpha _0.$

In the following results, we require that $\frac{f_0}{f_{\infty }}\in L^2(\mathbb {R}^{2n}, f_{\infty })$ which implies $f_0\in L^{1}(\mathbb {R}^{2d})$ because of the Hölder inequality $\int _{\mathbb {R}^{2n}}f_0 dxdv\le \sqrt{(\int _{\mathbb {R}^{2n}}\frac{f^2_0}{f_{\infty }}dxdv)(\int _{\mathbb {R}^{2n}}f_{\infty }dxdv)}$ and $\int _{\mathbb {R}^{2n}} f_{\infty }dxdv=1.$ We now state our first result, i.e. exponential decay of a functional that is a linear combination of the weighted $L^2$-norm and a Fisher information-type functional:

### Theorem 2.3

Let *V* be a $C^{\infty }$ potential in $\mathbb {R}^n$ satisfying Assumptions 2.1 and 2.2. Let $C_{PI},$ *c*,  $\tau ,$ and $\alpha _0$ be the constants in (5), (6), and (8). Suppose the initial data $f_0$ satisfies $\displaystyle \frac{f_0}{f_{\infty }}\in H^1(\mathbb {R}^{2n}, f_{\infty })$ and $\displaystyle \int _{\mathbb {R}^{2n}} \nabla ^T_v \left( \frac{f_0}{f_{\infty }}\right) \frac{\partial ^2V}{\partial x^2}\nabla _v \left( \frac{f_0}{f_{\infty }}\right) f_{\infty }dxdv<\infty .$ Then there are explicitly computable constants $C>0$ and $\lambda >0$ (independent of $f_0$) such that the solution *f*(*t*) of (1) satisfies

9 $$\begin{aligned}&\int _{\mathbb {R}^{2n}}\left( \frac{f(t)}{f_{\infty }}-1\right) ^2 f_{\infty }dxdv +\int _{\mathbb {R}^{2n}}\left| \nabla _{x}\left( \frac{f(t)}{f_{\infty }}\right) \right| ^2 f_{\infty }dxdv \nonumber \\&\quad +\int _{\mathbb {R}^{2n}}\nabla ^T_{v}\left( \frac{f(t)}{f_{\infty }}\right) \left( \frac{\partial ^2 V}{\partial x^2} +(1-\alpha _0)I\right) \nabla _{v}\left( \frac{f(t)}{f_{\infty }}\right) f_{\infty } dxdv \nonumber \\&\le C e^{-2\lambda t} \left[ \int _{\mathbb {R}^{2n}}\left( \frac{f_0}{f_{\infty }}-1\right) ^2 f_{\infty }dxdv+\int _{\mathbb {R}^{2n}}\left| \nabla _{x}\left( \frac{f_0}{f_{\infty }}\right) \right| ^2 f_{\infty }dxdv\right. \nonumber \\&\quad + \left. \int _{\mathbb {R}^{2n}}\nabla ^T_{v}\left( \frac{f_0}{f_{\infty }}\right) \left( \frac{\partial ^2 V}{\partial x^2}+ (1-\alpha _0) I\right) \nabla _{v}\left( \frac{f_0}{f_{\infty }}\right) f_{\infty }dxdv\right] \end{aligned}$$

for all $t\ge 0.$ Moreover, we have:

- if $$\displaystyle \alpha _0>\frac{\nu ^2}{4},\, \, \, c\le -\frac{\nu ^2}{4}, $$ then $$\displaystyle 2\lambda ={\nu -\tau };$$
- if $\displaystyle c=-\alpha _0=-\frac{\nu ^2}{4},$ then $\displaystyle 2\lambda ={\nu -\tau -\varepsilon }$ for any $\displaystyle \varepsilon \in (0,\nu -\tau );$
- if $$\displaystyle c>-\frac{\nu ^2}{4}, \,\, c+2\alpha _0> \frac{\nu ^2}{4},$$ then
  $$\begin{aligned} \displaystyle 2\lambda ={\left\{ \begin{array}{ll} \displaystyle \nu -\tau -\frac{c+\frac{\nu ^2}{4}}{\sqrt{c+\alpha _0}} &{} \text { if } \nu -\tau \ge A^{-1}_1+\frac{c+\frac{\nu ^2}{4}}{\sqrt{c+\alpha _0}} \\ \displaystyle \frac{(\nu -\tau )\sqrt{c+\alpha _0}-\Big (c+\frac{\nu ^2}{4}\Big ) \Big (\sqrt{1+s_1^2}-s_1\Big )}{{\sqrt{c+\alpha _0}+A_1s_1\Big (c+\frac{\nu ^2}{4}}\Big ) } &{} \text { if } \nu -\tau < A^{-1}_1+\frac{c+\frac{\nu ^2}{4}}{\sqrt{c+\alpha _0}} \end{array}\right. }, \end{aligned}$$
  where $$ \displaystyle A_1:=\frac{1+ \frac{\nu ^2}{4}+c+\alpha _0+ \sqrt{\Big ( \frac{\nu ^2}{4}+c+\alpha _0-1\Big )^2+\nu ^2}}{2\sigma C_{PI}},$$
  $$\begin{aligned} s_1:={\left\{ \begin{array}{ll} \frac{A^2_1\big (c+\frac{\nu ^2}{4}\big )^2-c-\alpha _0}{2A_1\big (c+\frac{\nu ^2}{4}\big ) \sqrt{c+\alpha _0}}&{} \text { if } (\nu -\tau )A_1=2 \\ \frac{1}{\nu -\tau }\left[ \left| \frac{(\nu -\tau )A_1-1}{(\nu -\tau )A_1-2}\right| \sqrt{\frac{\big (c+\frac{\nu ^2}{4}\big )^2}{c+\alpha _0} +2(\nu -\tau )A^{-1}_1-(\nu -\tau )^2}-\frac{c+\frac{\nu ^2}{4}}{((\nu -\tau )A_1-2)\sqrt{c+\alpha _0}}\right] &{} \text { if } (\nu -\tau )A_1\ne 2 \end{array}\right. }; \end{aligned}$$
- if $$\displaystyle c >-\frac{\nu ^2}{4}, \,\, c+2\alpha _0\le \frac{\nu ^2}{4},$$ then
  $$\begin{aligned} \displaystyle 2\lambda ={\left\{ \begin{array}{ll}\displaystyle \nu -\tau -\sqrt{{\nu ^2}-4\alpha _0} &{} \text { if } \nu -\tau \ge A^{-1}_2+\sqrt{{\nu ^2}-4\alpha _0} \\ \displaystyle \frac{\nu -\tau -\sqrt{{\nu ^2}-4\alpha _0} \Big (\sqrt{1+s_2^2}-s_2\Big )}{1+A_2s_2\sqrt{{\nu ^2}-4\alpha _0} } &{} \text { if } \nu -\tau < A^{-1}_2+\sqrt{{\nu ^2}-4\alpha _0} \end{array}\right. }, \end{aligned}$$
  where $$\displaystyle A_2:=\frac{1+ \frac{\nu ^2}{2}-\alpha _0+ \sqrt{\Big ( \frac{\nu ^2}{2}-\alpha _0-1\Big )^2+\nu ^2}}{2\sigma C_{PI}}, $$
  $$\begin{aligned} s_2:={\left\{ \begin{array}{ll} \frac{A^2_2 \big ({\nu ^2-4\alpha _0}\big )-1}{2A_2\sqrt{{\nu ^2}-4\alpha _0}}&{} \text { if } (\nu -\tau )A_2=2 \\ \frac{1}{\nu -\tau }\left[ \left| \frac{(\nu -\tau )A_2-1}{(\nu -\tau )A_2-2}\right| \sqrt{{{\nu ^2} -4\alpha _0}+2(\nu -\tau )A^{-1}_2-(\nu -\tau )^2}-\frac{\sqrt{{\nu ^2}-4\alpha _0}}{((\nu -\tau )A_2-2)}\right] &{} \text { if } (\nu -\tau )A_2\ne 2 \end{array}\right. }; \end{aligned}$$
- if *V*(*x*) is a quadratic polynomial of *x* and $\frac{\partial ^2 V}{\partial x^2}$ is positive definite, then Assumptions 2.1 and 2.2 are satisfied with $\tau =0,$ $c=-\alpha _0$ [this rules out the conditions in the case of (c)]. Moreover, the decay rates $\lambda$ in (a) and (d) are sharp and, in the case of (d), $\nu \ge {A^{-1}_2}+\sqrt{{\nu ^2}-4\alpha _0}$ holds and so $2\lambda = \nu -\sqrt{{\nu ^2}-4\alpha _0}.$ In the case of (b), the decay rate $2\lambda =\nu -\varepsilon$ is sharp in the sense that (9) holds with the rate $2\lambda =\nu -\varepsilon$ for any small fixed $\varepsilon \in (0,\nu ),$ but it does not hold with the rate $2\lambda =\nu .$

### Remark 2.4

1. It is possible to make weaker regularity hypothesis on the potential *V*,  but we maintain the assumption that $V \in C^{\infty }$ to keep the presentation simple.
2. Since $\frac{\partial ^2 V}{\partial x^2}+ (1-\alpha _0) I \ge I,$ (9) implies that the solution converges exponentially to the steady state in $H^1(\mathbb {R}^{2n}, f_{\infty }).$ If the eigenvalues of $\frac{\partial ^2 V}{\partial x^2}$ are uniformly bounded, then (9) is equivalent to the exponential decay of the solution to the steady state in $H^1(\mathbb {R}^{2n}, f_{\infty }).$ Due to the Poincaré inequality (5), the $L^2$-term on the right hand side of (9) could be omitted.
3. If *V* satisfies Assumption 2.2 with some constants $c\in \mathbb {R}$ and $\tau \in [0,\nu ),$ then *V* also satisfies Assumption 2.2 with any ${\tilde{c}}\ge c$ and ${\tilde{\tau }}\in [\tau ,\nu ).$ Therefore, these constants are not unique. But the exponential decay rate $\lambda$ obtained in Theorem 2.3 depends on the choice of *c* and $\tau .$ To obtain a better rate, one has to optimize $\lambda =\lambda (c,\tau )$ with respect to all *c* and $\tau$ satisfying Assumption 2.2.
4. In Theorem 2.3 (*b*),  the constant *C* in (9) depends on $\varepsilon ,$ and $C=C(\varepsilon )\rightarrow \infty$ as $\varepsilon \rightarrow 0.$
5. The highest exponential rate is $\frac{\nu }{2}$ which can be attained by the quadratic potentials *V* with $\frac{\partial ^2 V}{\partial x^2}\ge \frac{\nu ^2}{4}I.$

When *V* is a quadratic polynomial as in Theorem 2.3 (*e*),  we prove the following sharp estimates.

### Proposition 2.5

Let *V* be a quadratic polynomial and $\frac{\partial ^2 V}{\partial x^2}$ be positive definite. Let $\alpha _0>0$ be the smallest eigenvalue of $\frac{\partial ^2 V}{\partial x^2},$ then^2^

10 $$\begin{aligned} \sup _{1\ne \frac{f_0}{f_{\infty }} \in L^2(\mathbb {R}^d, f_{\infty })}\frac{||f(t)/f_{\infty }{-}1||_{L^2(\mathbb {R}^d, f_{\infty })}}{||f_0/f_{\infty }-1||_{L^2(\mathbb {R}^d, f_{\infty })}}\asymp {\left\{ \begin{array}{ll} e^{{-}\frac{\nu }{2}t},&{} \text { if } \alpha _0>\frac{\nu ^2}{4} \\ (1+ t)e^{{-}\frac{\nu }{2}t},&{} \text { if } \alpha _0=\frac{\nu ^2}{4}\\ e^{-{\frac{\nu -\sqrt{\nu ^2-4\alpha _0}}{2}t}},&{} \text { if } \alpha _0<\frac{\nu ^2}{4} \end{array}\right. } \, \, \, \, \, \text { as } \, \, t\rightarrow \infty .\nonumber \\ \end{aligned}$$

We shall use this proposition to prove the sharpness of the decay rates in Theorem 2.3 (*e*). When *V* is a quadratic polynomial and $-\alpha _0=-\frac{\nu ^2}{4}{=}{:}c,$ Theorem 2.3 (*e*) shows that the decay in (9) can be $e^{-(\nu -\varepsilon )t}$ for any small fixed $\varepsilon \in (0,\nu ),$ but it can not be $e^{-\nu t}.$ In this case, it is natural to expect a decay between $e^{-\nu t}$ and $e^{-(\nu -\varepsilon )t}:$ Proposition 2.5 shows that this is indeed the case for the square of the $L^2$-norm, with the decay $(1+ t)^2e^{-{\nu }t}.$ But an analogous extension of this result for the functional on the left hand side of (9) (i.e., to replace the term $C e^{-(\nu -\varepsilon ) t}$ with $C(1+t)^2e^{-\nu t}$ ) has not been obtained so far.

### Remark 2.6

Under assumptions of Proposition 2.5, we can construct special solutions $f_s(t)$ (see [3, Section 6]) which satisfy

$$\begin{aligned} \frac{||f_s(t)/f_{\infty }-1||_{L^2(\mathbb {R}^d, f_{\infty })}}{||f_0/f_{\infty }-1||_{L^2(\mathbb {R}^d, f_{\infty })}}\asymp {\left\{ \begin{array}{ll} e^{-\frac{\nu }{2}t},&{} \text { if } \alpha _0>\frac{\nu ^2}{4} \\ (1+ t)e^{-\frac{\nu }{2}t},&{} \text { if } \alpha _0=\frac{\nu ^2}{4}\\ e^{-{\frac{\nu -\sqrt{\nu ^2-4\alpha _0}}{2}t}},&{} \text { if } \alpha _0<\frac{\nu ^2}{4} \end{array}\right. } \, \, \, \, \, \text { as } \, \, t\rightarrow \infty . \end{aligned}$$

Our next result is about the estimates on the hypoelliptic regularization.

### Theorem 2.7

Assume *V* is a $C^{\infty }$ potential on $\mathbb {R}^n$ and there are constants $c \in \mathbb {R}$ and $\tau \ge 0$ such that the matrix (6) is positive semi-definite for all $x \in \mathbb {R}^n.$ Suppose the initial data $f_0$ satisfies $\displaystyle \int _{\mathbb {R}^{2n}} \left( \frac{f_0}{f_{\infty }}-1 \right) ^2 \left( \left| \left| \frac{\partial ^2V}{\partial x^2}\right| \right| ^2+1\right) f_{\infty }dxdv<\infty .$ Then, for any $t_0>0,$ there are explicitly computable constants $C_1=C_1(t_0)>0$ and $C_2=C_2(t_0)>0$ (independent of $f_0$) such that the inequalities

11 $$\begin{aligned} \displaystyle \int _{\mathbb {R}^{2n}}\left| \nabla _x \left( \frac{f(t)}{f_{\infty }}\right) \right| ^2f_{\infty } dx dv \le \frac{C_1}{t^{3}} \int _{\mathbb {R}^{2n}} \left( \frac{f_0}{f_{\infty }} -1\right) ^2 \left( \left| \left| \frac{\partial ^2V}{\partial x^2}\right| \right| ^2+1\right) f_{\infty }dxdv \end{aligned}$$

and

12 $$\begin{aligned}&\displaystyle \int _{\mathbb {R}^{2n}}\nabla _v^T \left( \frac{f(t)}{f_{\infty }}\right) \left( \frac{\partial ^2V}{\partial x^2}+(1-\alpha _0)I\right) \nabla _v \left( \frac{f(t)}{f_{\infty }}\right) f_{\infty } dx dv \nonumber \\&\le \frac{C_2}{t}\int _{\mathbb {R}^{2n}} \left( \frac{f_0}{f_{\infty }} -1\right) ^2 \left( \left| \left| \frac{\partial ^2V}{\partial x^2}\right| \right| ^2+1\right) f_{\infty }dxdv \end{aligned}$$

hold for all $t \in (0,t_0].$

In Theorem 2.3 we assumed that the initial data $f_0/f_{\infty }$ is in $H^1(\mathbb {R}^d, f_{\infty }).$ If we use the estimates in Theorem 2.7, this condition can be relaxed:

### Corollary 2.8

Let *V* be a $C^{\infty }$ potential in $\mathbb {R}^n$ satisfying Assumptions 2.1 and 2.2. Suppose the initial data $f_0$ satisfies $\displaystyle \int _{\mathbb {R}^{2n}} \left( \frac{f_0}{f_{\infty }} -1\right) ^2 \left( \left| \left| \frac{\partial ^2V}{\partial x^2}\right| \right| ^2+1\right) f_{\infty }dxdv<\infty .$ Then, for any $t_0>0,$ there is an explicitly computable constant $C=C(t_0)>0$ (independent of $f_0$) such that

13 $$\begin{aligned}&\int _{\mathbb {R}^{2n}}\left( \frac{f(t)}{f_{\infty }}-1\right) ^2 f_{\infty }dxdv +\int _{\mathbb {R}^{2n}}\left| \nabla _{x}\left( \frac{f(t)}{f_{\infty }}\right) \right| ^2 f_{\infty }dxdv \nonumber \\&\qquad +\int _{\mathbb {R}^{2n}}\nabla ^T_{v}\left( \frac{f(t)}{f_{\infty }}\right) \left( \frac{\partial ^2 V}{\partial x^2}+(1-\alpha _0)I\right) \nabla _{v}\left( \frac{f(t)}{f_{\infty }}\right) f_{\infty } dxdv \nonumber \\&\quad \le C e^{-2\lambda t} \int _{\mathbb {R}^{2n}} \left( \frac{f_0}{f_{\infty }} -1\right) ^2 \left( \left| \left| \frac{\partial ^2V}{\partial x^2}\right| \right| ^2+1\right) f_{\infty }dxdv \end{aligned}$$

holds for all $t\ge t_0$ with $\lambda$ defined in Theorem 2.3.

### Remark 2.9

1. In contrast to Theorem 2.3, Theorem 2.7 holds even if the Poincaré inequality (5) is not satisfied by $f_{\infty }.$ Also, $\tau$ can be larger than $\nu .$
2. The exponents of *t* in (11) and (12) are optimal when *V* is a quadratic polynomial (see [32, Appendix A]).

To illustrate our result, we present concrete examples of potentials *V* satisfying our Assumptions 2.1 and 2.2:

### Example 2.10

(Polynomial confining potentials) (*a*) As mentioned in Theorem 2.3, if $V(x)=\frac{{x}^T M^{-1} x}{2}+p\cdot x+q, \,$ $x\in \mathbb {R}^n$ with a positive definite covariance matrix $M^{-1}\in \mathbb {R}^{n\times n},$ a constant vector $p\in \mathbb {R}^n$ and a constant $q\in \mathbb {R},$ the convergence rate is

$$\begin{aligned} \lambda ={\left\{ \begin{array}{ll} \frac{\nu }{2},&{} \text { if } \alpha _0>\frac{\nu ^2}{4} \, \, \, \, \,\, \, \, \, \, \,\, \, \, \, \, \,\, \, \, \, \, \,\, \, \, \, \, \,\, \, \, \, \, \,\, \, \, \, \, \,\, \, \, \, \, \,\, \, \, \, \, \, \text { (case (a))}\\ \frac{ \nu -\varepsilon }{2},&{} \text { if } \alpha _0=\frac{\nu ^2}{4}, \text { for any } \varepsilon \in (0,\nu ) \, \, \, \, \text { (case (b))}\\ \frac{\nu -\sqrt{\nu ^2-4\alpha _0}}{2},&{} \text { if } \alpha _0<\frac{\nu ^2}{4} \,\, \, \, \, \,\, \, \, \, \, \,\, \, \, \, \, \,\, \, \, \, \, \,\, \, \, \, \, \,\, \, \, \, \, \,\, \, \, \, \, \,\, \, \, \, \, \,\, \, \, \, \, \, \text { (case (d))} \end{array}\right. }, \end{aligned}$$

and it is sharp for $\alpha _0\ne \frac{\nu ^2}{4},$ where $\alpha _0$ is the smallest eigenvalue of $M^{-1}$ (see Theorem 2.3 (*e*)).

(*b*) More generally, we consider potentials of the form

$$\begin{aligned} V(x)=r|x|^{2k}+V_0(x) \end{aligned}$$

where $r>0,$ $k \in \mathbb {N}$ and $V_0:\mathbb {R}^n \rightarrow \mathbb {R}$ is a polynomial of degree $j<2k.$ Since we have already considered quadratic potentials, we assume $k\ge 2.$*V* satisfies the Poincaré inequality (5); this can be proven, for example, by showing that *V* satisfies one of the sufficient conditions given in [6, Corollary 1.6]. Concerning Assumption 2.2’ we have

$$\begin{aligned} r\frac{\partial ^2 |x|^{2k}}{\partial x^2}=2kr|x|^{2k-2}I+2k(2k-2)r|x|^{2k-4} \begin{pmatrix} x_1^2&{} x_1x_2&{}...&{}x_1x_n\\ x_1x_2&{}x^2_2&{}...&{}x_2x_n\\ ...&{}...&{}...&{}...\\ x_1x_n&{}x_2 x_n&{}...&{}x_n^2\\ \end{pmatrix}\ge 2kr|x|^{2k-2}I. \end{aligned}$$

Since $$V_0 $$ has degree $$j<2k,$$ there is a constant $$A>0$$ such that

$$\begin{aligned} -A\big (1+|x|^{2k-3}\big )I\le \frac{\partial ^2 V_0(x)}{\partial x^2}\le A\big (1+|x|^{2k-3}\big )I. \end{aligned}$$

Therefore, we can estimate

14 $$\begin{aligned} \frac{\partial ^2 V(x)}{\partial x^2}\ge \left( 2kr|x|^{2k-2}-A|x|^{2k-3}-A\right) I. \end{aligned}$$

We also observe that there exists a positive constant *B* such that

$$\begin{aligned} -B\big (1+|x|^{2k-3}\big )I\le \frac{\partial ^2(\partial _{x_{i}} V(x))}{\partial x^2}\le B\big (1+|x|^{2k-3}\big )I \end{aligned}$$

for all $$ i \in \{1,...,n\}.$$ (14) shows that the smallest eigenvalue of $$\frac{\partial ^2 V(x)}{\partial x^2}$$ satisfies $$\alpha (x)\ge 2kr|x|^{2k-2}-A|x|^{2k-3}-A.$$ Since $$ 2kr|x|^{2k-2}-A|x|^{2k-3}-A$$ grows faster than $$B(1+|x|^{2k-3})$$ as $$|x| \rightarrow \infty ,$$ there are constants *c* and $$\tau \in [0,\nu )$$ such that (7) is satisfied. Thus, Theorem 2.3 applies to this type of potentials. In particular, it applies to double-well potentials of the form $$V(x)=r_1|x|^4-r_2|x|^2, \,\, \, r_1,r_2>0.$$

### Remark 2.11

1. Our decay and regularization results above extend those of [23], where a stronger assumption, i.e. $\partial ^{2}_{x_i x_j}V \in \bigcap _{p=1}^{\infty }W^{p,\infty }(\mathbb {R}^n)$ for all $i,j \in \{1,...,n\},$ was made. By contrast, we did not require the boundedness of the second and higher derivatives of *V*.
2. Most of the previous works on the exponential convergence $f(t)\rightarrow f_{\infty }$ as $t\rightarrow \infty$ (e.g. [9, 10, 14, 15, 20, 31, 32]) used the growth condition (4) to get some weighted Poincaré type inequalities (see [31, Lemma A.24]), which are crucial in these works—and additional to the Poincaré inequality (5). Our technique is rather different, based on construction of appropriate state dependent matrices and state dependent matrix inequalities so that the (modified) dissipation functional (see (20) below) decays exponentially.
3. Most of the previous methods for proving the exponential convergence do not give an accurate decay rate, $\lambda$ is typically much too small there (see [31, Section 7.2], [20, Section 1.4]). For example, in [31, Section 7.2], the exponential decay rate $\lambda =\frac{1}{40}$ was obtained for $V(x)=\frac{|x|^2}{2}$ and $\nu =\sigma =1.$ Since our decay rates are sharp for quadratic potentials, in this setting, the true rate $\lambda =\frac{1}{2}$ is given by Theorem 2.3 (*a*) and (*e*).

## Modified Entropy Methods for Degenerate Fokker–Planck Equations

We first consider the following degenerate and non-symmetric Fokker–Planck equation [1, 2]:

15 $$\begin{aligned} {\left\{ \begin{array}{ll} \partial _t f=\text {div}(D\nabla f+(D+R)\nabla E f), \, \, \xi \in \mathbb {R}^d, \, t>0,\\ f(t=0)=f_0\in L_+^1(\mathbb {R}^d),\,\, \int _{\mathbb {R}^d}f_0 \,d\xi =1 \end{array}\right. } \end{aligned}$$

where $D\in \mathbb {R}^{d\times d}$ is a constant, symmetric, positive semi-definite $(\text {rank}(D)< d)$ matrix, $R\in \mathbb {R}^{d\times d}$ is a constant skew-symmetric matrix. $E:\mathbb {R}^d\rightarrow \mathbb {R}$ is a function which only depends on the state variable $\xi .$ We assume that *E* is confining (i.e. $E(\xi )\rightarrow \infty$ for $|\xi |\rightarrow \infty$) and smooth enough so that (15) has a unique and smooth solution. If *E* grows fast enough, (15) has a normalized steady state $f_{\infty }=c_E e^{-E}, \, c_{E}>0.$ The weak maximum principle for degenerate parabolic equations [25] can be applied to (15) and we can prove that $f(t,\xi )\ge 0$ for all $t>0, \, \xi \in \mathbb {R}^d.$ The divergence structure implies that the initial mass is conserved and $f(t,\cdot )$ describes the evolution of a probability density

$$\begin{aligned} \int _{\mathbb {R}^d}f(t,\xi )d\xi =\int _{\mathbb {R}^d}f_0(\xi )d\xi =1, \, \, \, \forall t\ge 0. \end{aligned}$$

We are interested in the large-time behavior of the solution, in particular, when $\text {rank}(D)$ is less than the dimension *d*. When *D* is positive definite (rank$(D)=d$), the large time behavior and exponential convergence have been studied comprehensively (see [2, 4, 7]). One of the well-known conditions which provides the exponential decay of the solution to the steady state is called *the Bakry–Emery condition* (see (16) below) leading to:

### Theorem 3.1

[2, Theorem 2.6] Assume $\displaystyle \int _{\mathbb {R}^d}\left( \frac{f_0}{f_{\infty }}-1\right) ^2 f_{\infty }d\xi <\infty$ and

16 $$\begin{aligned} \exists {\lambda }>0 \, \text { such that } \,{\frac{\partial ^2 E}{\partial \xi ^2}(I+RD^{-1})+ \left( \frac{\partial ^2 E}{\partial \xi ^2}(I+RD^{-1})\right) ^T\ge \lambda D^{-1}}, \, \, \,\forall \xi \in \mathbb {R}^d.\nonumber \\ \end{aligned}$$

Then

$$\begin{aligned} \int _{\mathbb {R}^d}\left( \frac{f(t)}{f_{\infty }}-1\right) ^2 f_{\infty }d\xi \le e^{-2\lambda t}\int _{\mathbb {R}^d}\left( \frac{f_0}{f_{\infty }}-1\right) ^2 f_{\infty }d\xi . \end{aligned}$$

To prove the theorem above, one considers the time derivative of the $L^2$-norm and we see that it decreases

17 $$\begin{aligned} \frac{d}{dt}\int _{\mathbb {R}^d}\left( \frac{f(t)}{f_{\infty }}{-}1\right) ^2 f_{\infty }d\xi {=}{-}2\int _{\mathbb {R}^d}\nabla ^T\left( \frac{f}{f_{\infty }}\right) {D} \nabla \left( \frac{f}{f_{\infty }}\right) f_{\infty }d\xi {=}:{-}I(f(t)|f_{\infty })\le 0.\nonumber \\ \end{aligned}$$

$I(f(t)|f_{\infty })$ is called the dissipation functional and since *D* is positive definite it vanishes if and only if $f=f_{\infty }.$ It can be proven that, under the Bakry–Emery condition,

18 $$\begin{aligned} \frac{d}{dt} I(f(t)|f_{\infty })\le -2\lambda I(f(t)|f_{\infty }). \end{aligned}$$

Integrating this inequality from $(t,\infty )$ and using the convergences $I(f(t)|f_{\infty })\rightarrow 0$ and $\displaystyle \int _{\mathbb {R}^d}\left( \frac{f(t)}{f_{\infty }}-1\right) ^2 f_{\infty }d\xi \rightarrow 0$ as $t \rightarrow \infty ,$ it follows that

19 $$\begin{aligned} \frac{d}{dt}\int _{\mathbb {R}^d}\left( \frac{f(t)}{f_{\infty }}-1\right) ^2 f_{\infty }d\xi \le -2\lambda \int _{\mathbb {R}^d}\left( \frac{f(t)}{f_{\infty }}-1\right) ^2 f_{\infty }d\xi \end{aligned}$$

and, by Grönwall’s lemma, we get the desired result.

When *D* is only positive semi-definite, i.e. rank$(D)<d,$ one observes that $I(f(t)|f_{\infty })$ may vanish for certain probability densities $f\ne f_{\infty }.$ Hence the inequalities (18) and (19) will not hold in general. Since the above problems stem from the singularity of *D*,  one can modify the dissipation function and define a modified dissipation functional (see also [1, 3])

20 $$\begin{aligned} S(f):=2\int _{\mathbb {R}^d}\nabla ^T_{\xi }\left( \frac{f}{f_{\infty }}\right) {P(\xi )} \nabla _{\xi }\left( \frac{f}{f_{\infty }}\right) f_{\infty }d\xi \end{aligned}$$

where $P:\mathbb {R}^d\rightarrow \mathbb {R}^{d \times d}$ is a symmetric positive definite matrix which will be chosen later. Extending the approach of [1, 3], we allow the matrix *P* here to depend on $\xi \in \mathbb {R}^d.$ Our goal is to derive a differential inequality similar to (18) (like the dissipation functional satisfied for non-degenerate equations), i.e.

21 $$\begin{aligned} \frac{d}{dt}S(f(t))\le -2\lambda S(f(t)),\, \, \, \, \end{aligned}$$

for some $\lambda >0$ and a “good” choice of the matrix *P*. If this holds true, we would obtain

$$\begin{aligned} S(f(t))\le S(f_0)e^{-2\lambda t}. \end{aligned}$$

If we can choose such $P=P(\xi )\ge \eta I$ for some $\eta >0$ and all $\xi \in \mathbb {R}^d$, under the validity of the Poincaré inequality (5) for $f_{\infty }(\xi )=c_E e^{-E(\xi )}$ (where $$\begin{pmatrix} x\\ v \end{pmatrix}$$ in (5) is replaced with $\xi$) we have

$$\begin{aligned} \int _{\mathbb {R}^d}\left( \frac{f(t)}{f_{\infty }}-1\right) ^2 f_{\infty }d\xi \le \frac{1}{C_{PI}} \int _{\mathbb {R}^{d}} \left| \nabla _{\xi } \left( \frac{f(t)}{f_{\infty }}\right) \right| ^2f_{\infty }d\xi \le \frac{1}{2C_{PI}\eta }S(f(t)), \, \, \, \end{aligned}$$

which implies the exponential decay of the $L^2$-norm

$$\begin{aligned} \int _{\mathbb {R}^d}\left( \frac{f(t)}{f_{\infty }}-1\right) ^2 f_{\infty }d\xi \le \frac{1}{2 C_{PI}\eta }S(f_0) e^{-2\lambda t}. \end{aligned}$$

More generally, since the quadratic entropy is also a decreasing function of time *t*, instead of proving (21), we can consider the functional

22 $$\begin{aligned}&\Phi (f(t)){:}{=}\gamma \int _{\mathbb {R}^d}\left( \frac{f(t)}{f_{\infty }}-1\right) ^2 f_{\infty }d\xi +S(f(t))\nonumber \\&\qquad \qquad \,\, = \gamma \int _{\mathbb {R}^d}\left( \frac{f(t)}{f_{\infty }}-1\right) ^2 f_{\infty }d\xi +2\int _{\mathbb {R}^d}\nabla ^T\left( \frac{f}{f_{\infty }}\right) {P(\xi )} \nabla \left( \frac{f}{f_{\infty }}\right) f_{\infty }d\xi \end{aligned}$$

and choose a suitable parameter $\gamma \ge 0$ and a matrix *P* such that

23 $$\begin{aligned} \frac{d \Phi (f(t))}{dt}\le -2\lambda \Phi (f(t))\le 0 \end{aligned}$$

for some $\lambda >0.$ This idea and method were successfully applied in [3] to (15) when the potential *E* is quadratic.

We shall apply this method to the kinetic Fokker–Planck equation with non-quadratic *V*(*x*). First, we denote $$\xi {:}{=}\begin{pmatrix} x\\ v \end{pmatrix} \in \mathbb {R}^{2n},$$ $E(\xi ):=\frac{\nu }{\sigma }[V(x)+\frac{|v|^2}{2}],\, f_{\infty }=e^{-E}.$ Then the kinetic Fokker–Planck equation (1) can be written in the form of (15),

24 $$\begin{aligned} \partial _t f=\text {div}_{\xi }(D\nabla _{\xi } f+(D+R)\nabla _{\xi } E f) \end{aligned}$$

with

25 $$\begin{aligned} D=\begin{pmatrix} 0&{}0\\ 0&{}\sigma I \end{pmatrix} \in \mathbb {R}^{2n \times 2n} \, \, \, \, \, \, \text {and} \, \, \, \, \, \, R=\frac{\sigma }{\nu }\begin{pmatrix} 0&{}-I\\ I&{}0 \end{pmatrix} \in \mathbb {R}^{2n \times 2n}. \end{aligned}$$

The rank of the diffusion matrix *D* is $n<d=2n.$ Thus, (1) is both non-symmetric and degenerate and the arguments above apply to the equation.

We will develop a modified entropy method. We will choose $\xi$-dependent matrix *P* in the modified dissipation functional (20) so that (23) holds and $\lambda >0$ is as large as possible.

We also mention that when the potential *E* is quadratic in (15), the question about the long time behavior can be reduced to an ODE problem:

### Theorem 3.2

Let $0 \ne D \in \mathbb {R}^{d\times d}$ be positive semi-definite, $R\in \mathbb {R}^{d\times d}$ be skew-symmetric and $\mathbb {R}^{d} \ni \xi \rightarrow E(\xi )=\frac{{\xi }^T K^{-1} \xi }{2}$ for some positive definite matrix *K*. Assume $(D+R)K^{-1}$ is positive stable and there is no non-trivial subspace of $\text {Ker }D$ which is invariant under $K^{-1}(D-R).$ If *f* is the solution of (15) and $\xi (t)\in \mathbb {R}^d$ is the solution of the ODE ${\dot{\xi }}(t)=-K^{-\frac{1}{2}}(D+R)K^{-\frac{1}{2}}\xi$ with initial datum $\xi (0)=\xi _0,$ then

26 $$\begin{aligned} \sup _{1\ne \frac{f_0}{f_{\infty }} \in L^2(\mathbb {R}^d, f_{\infty })}\frac{||f(t)/f_{\infty }-1||_{L^2(\mathbb {R}^d, f_{\infty })}}{||f_0/f_{\infty }-1||_{L^2(\mathbb {R}^d, f_{\infty })}}=\sup _{0\ne \xi _0\in \mathbb {R}^d}\frac{||\xi (t)||_2}{||\xi _0||_2}, \, \, \, t\ge 0. \end{aligned}$$

### Proof

See [5, Theorem 3.4]. $$\square $$

One consequence of Theorem 3.2 is that the decay estimate of the ODE-solution carries over to the corresponding Fokker–Planck equation.

## The Choice of the Matrix P

For future reference (in the proof of Theorem 2.7) we shall now also allow the matrix *P* to be time dependent. Hence we shall next consider the generalized functional

$$\begin{aligned} S(t,f):=2\int _{\mathbb {R}^d}\nabla ^T_{\xi }\left( \frac{f}{f_{\infty }}\right) {P(t,\xi )} \nabla _{\xi }\left( \frac{f}{f_{\infty }}\right) f_{\infty }d\xi . \end{aligned}$$

The following lemmas will play a crucial role in our arguments.

### Lemma 4.1

Let $P:{[0, \infty )\times \mathbb {R}^{2n}}\rightarrow \mathbb {R}^{2n \times 2n}$ be smooth and *f* be the solution of (1), then

27 $$\begin{aligned} \frac{d}{dt} {S(t,f(t))}&=-4\sigma \int _{\mathbb {R}^{2n}}\left\{ \sum _{i=1}^n (\partial _{v_i}u)^TP\partial _{v_i}u\right\} f_{\infty }dxdv\nonumber \\&\quad -2\int _{\mathbb {R}^{2n}} u^T\left\{ QP+PQ^T\right\} u f_{\infty }dxdv\nonumber \\&\quad -2\int _{\mathbb {R}^{2n}} u^T\left\{ [\nabla _x V\cdot \nabla _v-v\cdot \nabla _x+\nu v\cdot \nabla _v-\sigma \Delta _v-{\partial _t}]P\right\} u f_{\infty }dxdv, \end{aligned}$$

where $u{:}{=}\nabla _{x,v} \left( \frac{f}{f_{\infty }}\right) ,$ $$Q=Q(x){:}{=}\begin{pmatrix} 0&{}I\\ -\frac{\partial ^2 V(x)}{\partial x^2}&{}\nu I \end{pmatrix},$$ and $[\nabla _x V\cdot \nabla _v-v\cdot \nabla _x+\nu v\cdot \nabla _v-\sigma \Delta _v {-\partial _t}]$ denotes a scalar differential operator that is applied to each element of the matrix $P=P(t,x,v)$.

### Proof

We denote $\displaystyle u_1{:}{=}\nabla _x\left( \frac{f}{f_{\infty }}\right) ,$ $\displaystyle u_2{:}{=}\nabla _v\left( \frac{f}{f_{\infty }}\right) ,$ then $u_1$ and $u_2$ satisfy

$$\begin{aligned}{} & {} \partial _t u_1=\sigma \Delta _v u_1-\nu \sum _{i=1}^n v_i \partial _{v_i}u_1+\sum _{i=1}^n \partial _{x_i} V \partial _{v_i} u_1-\sum _{i=1}^n v_i \partial _{x_i} u_1+\frac{\partial ^2 V}{\partial x^2}u_2,\\{} & {} \partial _t u_2=\sigma \Delta _v u_2-\nu \sum _{i=1}^n v_i \partial _{v_i}u_2+\sum _{i=1}^n \partial _{x_i} V \partial _{v_i} u_2-\sum _{i=1}^n v_i \partial _{x_i} u_2-u_1-\nu u_2. \end{aligned}$$

These equations can be written with respect to $$u=\begin{pmatrix} u_1\\ u_2 \end{pmatrix}: $$

$$\begin{aligned} \partial _t u=\sigma \Delta _v u-\nu \sum _{i=1}^n v_i \partial _{v_i}u+\sum _{i=1}^n \partial _{x_i} V \partial _{v_i} u-\sum _{i=1}^n v_i \partial _{x_i} u-Q^Tu. \end{aligned}$$

It allows us to compute the time derivative of the modified dissipation functional

28 $$\begin{aligned}&\frac{d}{dt} {S(t,f(t))}=4\int _{\mathbb {R}^{2n}} u^T P \partial _t u f_{\infty }dxdv+{2\int _{\mathbb {R}^{2n}} u^T \partial _t P u f_{\infty }dxdv}\nonumber \\&\quad = 4\sigma \int _{\mathbb {R}^{2n}} u^T P \Delta _v u f_{\infty }dxdv-4\nu \sum _{i=1}^n\int _{\mathbb {R}^{2n}} u^T P \partial _{v_i}u v_i f_{\infty }dxdv \nonumber \\&\qquad + 4\sum _{i=1}^n \int _{\mathbb {R}^{2n}} u^TP \partial _{v_i} u \partial _{x_i} V f_{\infty }dxdv-4\sum _{i=1}^n\int _{\mathbb {R}^{2n}} u^TP \partial _{x_i}u v_i f_{\infty }dxdv \nonumber \\&\qquad -2\int _{\mathbb {R}^{2n}} u^T \{QP+PQ^T\}u f_{\infty }dxdv+{2\int _{\mathbb {R}^{2n}} u^T \partial _t P u f_{\infty }dxdv}. \end{aligned}$$

First, we consider the term in the second line of (28) and use $\partial _{v_i}f_{\infty }=-\frac{\nu }{\sigma }v_if_{\infty }:$

29 $$\begin{aligned}&4\sigma \sum _{i=1}^n\int _{\mathbb {R}^{2n}} u^TP \partial ^2_{v_iv_i} u f_{\infty }dxdv -4\nu \sum _{i=1}^n\int _{\mathbb {R}^{2n}} u^TP \partial _{v_i}u v_i f_{\infty }dxdv \nonumber \\&=-4\sigma \sum _{i=1}^n \int _{\mathbb {R}^{2n}}\partial _{v_i}u^TP\partial _{v_i}u f_{\infty }dxdv-4\sigma \sum _{i=1}^n\int _{\mathbb {R}^{2n}} u^T (\partial _{v_i}P)\partial _{v_i}u f_{\infty }dxdv. \end{aligned}$$

By integrating by parts the last term of (29) we obtain

$$\begin{aligned}&-4\sigma \sum _{i=1}^n\int _{\mathbb {R}^{2n}} u^T (\partial _{v_i}P)\partial _{v_i}u f_{\infty }dxdv\\&= 4\sigma \sum _{i=1}^n\int _{\mathbb {R}^{2n}} u^T (\partial _{v_i}P)\partial _{v_i}u f_{\infty }dxdv +4\sigma \sum _{i=1}^n\int _{\mathbb {R}^{2n}} u^T (\partial ^2_{v_i v_i}P)u f_{\infty }dxdv\\&-4\nu \sum _{i=1}^n\int _{\mathbb {R}^{2n}} u^T (\partial _{ v_i}P)u v_i f_{\infty }dxdv \end{aligned}$$

and we find

$$\begin{aligned}&-4\sigma \sum _{i=1}^n\int _{\mathbb {R}^{2n}} u^T (\partial _{v_i}P)\partial _{v_i}u f_{\infty }dxdv=2\sigma \int _{\mathbb {R}^{2n}} u^T (\Delta _v P)u f_{\infty }dxdv\\&-2\nu \sum _{i=1}^n\int _{\mathbb {R}^{2n}} u^T (v_i \partial _{ v_i}P)u f_{\infty }dxdv. \end{aligned}$$

If we use this equality in (29), we get

30 $$\begin{aligned}&4\sigma \int _{\mathbb {R}^{2n}} u^T P \Delta _v u f_{\infty }dxdv-4\nu \sum _{i=1}^n\int _{\mathbb {R}^{2n}} u^T P v_i \partial _{v_i}u f_{\infty }dxdv \nonumber \\&=-4\sigma \sum _{i=1}^n \int _{\mathbb {R}^{2n}}(\partial _{v_i}u)^TP\partial _{v_i}u f_{\infty }dxdv-2\int _{\mathbb {R}^{2n}} u^T \{[\nu v\cdot \nabla _v -\sigma \Delta _v] P\}u f_{\infty }dxdv. \end{aligned}$$

Next, we integrate by parts in the terms in the third line of (28):

31 $$\begin{aligned}&4\sum _{i=1}^n \int _{\mathbb {R}^{2n}} u^TP \partial _{v_i} u \partial _{x_i}V f_{\infty }dxdv \nonumber \\&= -4\sum _{i=1}^n \int _{\mathbb {R}^{2n}} u^TP \partial _{v_i} u \partial _{x_i} V f_{\infty }dxdv \nonumber \\&\quad -4\sum _{i=1}^n \int _{\mathbb {R}^{2n}} u^T(\partial _{v_i} P) u \partial _{x_i} V f_{\infty }dxdv+\frac{4\nu }{\sigma }\sum _{i=1}^n \int _{\mathbb {R}^{2n}} u^TP u \partial _{x_i} V v_i f_{\infty }dxdv, \end{aligned}$$

32 $$\begin{aligned}&\quad -4\sum _{i=1}^n\int _{\mathbb {R}^{2n}} u^TP \partial _{x_i}u v_i f_{\infty }dxdv \nonumber \\&=4\sum _{i=1}^n\int _{\mathbb {R}^{2n}} u^TP \partial _{x_i}u v_i f_{\infty }dxdv \nonumber \\&\quad +4\sum _{i=1}^n\int _{\mathbb {R}^{2n}} u^T(\partial _{x_i}P)uv_i f_{\infty }dxdv-\frac{4\nu }{\sigma }\sum _{i=1}^n\int _{\mathbb {R}^{2n}} u^TP u \partial _{x_i}V v_i f_{\infty }dxdv. \end{aligned}$$

(31) and (32) show that the third line of (28) equals

33 $$\begin{aligned}&-2\sum _{i=1}^n \int _{\mathbb {R}^{2n}} u^T(\partial _{v_i} P) u \partial _{x_i} V f_{\infty }dxdv+2\sum _{i=1}^n\int _{\mathbb {R}^{2n}} u^T(\partial _{x_i}P) u v_i f_{\infty }dxdv \nonumber \\&=-2\int _{\mathbb {R}^{2n}} u^T\{[\nabla _x V\cdot \nabla _v-v\cdot \nabla _x]P\} u f_{\infty }dxdv. \end{aligned}$$

Combining (28), (30), and (33) we obtain the statement (27). $\square$

### Remark 4.2

We give now a (formal) generalization of the above result (27) to Markovian evolution equations using the *Gamma calculus*, see, e.g., [8–10]:

First, let *L* be the generator of some Markovian evolution on $\mathbb {R}^d$ with corresponding invariant measure $f_\infty d\xi$. Let $P=P(\xi )$ be a smooth matrix function (but it does not have to be symmetric or positive definite). We define the first order bilinear form

$$\begin{aligned} \Gamma ^P(g,h){:}{=}\nabla _\xi g^T P \nabla _\xi h \end{aligned}$$

and

$$\begin{aligned} \Gamma ^P_2(g,h){:}{=}\frac{1}{2} \big (L \Gamma ^P(g,h)-\Gamma ^P(Lg,h)-\Gamma ^P(g,Lh)\big ). \end{aligned}$$

For a solution *h*(*t*) of $\partial _t h=Lh$, these definitions give

34 $$\begin{aligned} \frac{d}{dt} \Gamma ^P(h,h)=\Gamma ^P(Lh,h)+\Gamma ^P(h,Lh)=-2 \Gamma _2^P(h,h) + L \Gamma ^P(h,h),\qquad \forall \xi \in \mathbb {R}^d.\nonumber \\ \end{aligned}$$

We use $\Gamma ^P$ to define the modified dissipation functional

$$\begin{aligned} S(f) {:}{=}2\int _{\mathbb {R}^d} \Gamma ^P(h,h)\, f_\infty d\xi \quad \text { with } h=\frac{f}{f_\infty }. \end{aligned}$$

We obtain by integrating (34):

35 $$\begin{aligned} \frac{d}{dt} S(f(t)) = -4\int _{\mathbb {R}^d} \Gamma ^P_2(h,h)\, f_\infty d\xi , \end{aligned}$$

where we used that $\int _{\mathbb {R}^d} L\Gamma ^P(h,h)\, f_\infty d\xi =0$.

In particular, let *L* be the generator of the kinetic Fokker–Planck equation (3), and we recall that $$\xi {:}{=}\begin{pmatrix}x\\ v\end{pmatrix}.$$ Then, a straightforward (but lengthy) computation shows that

$$\begin{aligned}&2\Gamma _2^P(h,h)=2\sigma \sum _{i=1}^n (\partial _{v_i}u)^TP\partial _{v_i}u + u^T\left( QP+PQ^T\right) u + u^T (LP) u \\&\quad +2\sigma \sum _{i=1}^n (\partial _{v_i}u)^T(\partial _{v_i}P)u +4\sigma \sum _{i=1}^n u^T(\partial _{v_i}P)\partial _{v_i}u. \end{aligned}$$

One can check (by integrating by parts the term $4 \sigma \int _{\mathbb {R}^d} \sum _{i=1}^n u^T(\partial _{v_i}P)\partial _{v_i} u f_\infty d\xi$ in the right hand side of (35)) that (35) coincides with (27). Hence, (35) reproduces (27). But in contrast to (27), the preceding statement (34) is local in $\xi$ and therefore stronger.

The key question for using the modified entropy dissipation functional *S*(*f*) is how to choose the matrix *P*. To determine *P* we shall need the following algebraic result:

### Lemma 4.3

For any fixed matrix $\mathcal {Q}\in \mathbb {R}^{d\times d},$ let $\mu :=\min \{\textsf {Re}(\beta ): \beta \text { is an eigenvalue of } \mathcal {Q}\}.$ Let $\{\beta _m: 1\le m\le m_0\}$ be all the eigenvalues of $\mathcal {Q}$ with $\mu =\textsf {Re}(\beta ),$ only counting their geometric multiplicity.

(a) If $\beta _m$ is non-defective for all $m\in \{1,...,m_0\},$ then there exists a symmetric, positive definite matrix $P\in \mathbb {R}^{d\times d}$ with

$$\begin{aligned} \mathcal {Q} P+P\mathcal {Q}^T\ge 2\mu P. \end{aligned}$$

(b) If $\beta _m$ is defective for at least one $m\in \{1,...,m_0\},$ then for any $\varepsilon >0$ there exists a symmetric, positive definite matrix $P(\varepsilon )\in \mathbb {R}^{d\times d}$ with

$$\begin{aligned} \mathcal {Q}P(\varepsilon )+P(\varepsilon )\mathcal {Q}^T\ge 2(\mu -\varepsilon ) P(\varepsilon ). \end{aligned}$$

### Proof

See [3, Lemma 4.3]. $$\square $$

We consider the matrix function

36 $$\begin{aligned} Q(x){:}{=}\begin{pmatrix} 0&{}I\\ -\frac{\partial ^2 V(x)}{\partial x^2}&{}\nu I \end{pmatrix}, \, \, \, \, x \in \mathbb {R}^n, \end{aligned}$$

which appears in (27). We want to construct a symmetric positive definite matrix *P*(*x*) such that $Q(x)P(x)+P(x)Q^T(x)$ is positive definite and

$$\begin{aligned} Q(x)P(x)+P(x)Q^T(x)\ge 2\mu P(x) \end{aligned}$$

for some $\mu >0$ and for all $x \in \mathbb {R}^n.$ We recall

$$\begin{aligned} \displaystyle \alpha (x){:}{=}\min _{i\in \{1,..,n\} }\left\{ \alpha _i(x)\,: \,\alpha _i (x) \, \text { is an eigenvalue of } \frac{\partial ^2 V(x)}{\partial x^2}\right\} , \end{aligned}$$

$$\begin{aligned}{} & {} \displaystyle \alpha _0 {:}{=}\inf _{x \in \mathbb {R}^n}\alpha (x),\\{} & {} \mu {:}{=}\inf _{x \in \mathbb {R}^n, \,i\in \{1,..,n\}}\{\textsf {Re}(\beta _i(x)): \beta _i(x) \text { is an eigenvalue of } Q(x)\}. \end{aligned}$$

### Lemma 4.4

(1) The matrix *Q*(*x*) is positive stable at any fixed $x \in \mathbb {R}^n,$ if and only if $\frac{\partial ^2 V(x)}{\partial x^2}$ is positive definite.

(2) Let $\frac{\partial ^2 V(x)}{\partial x^2}$ be positive definite for some $x \in \mathbb {R}^n.$ Then:

- If $\alpha _0> \frac{\nu ^2}{4},$ then $\mu =\frac{\nu }{2}$ and there exists a symmetric positive definite matrix *P*(*x*) such that
  $$\begin{aligned} Q(x)P(x)+P(x)Q^T(x)=2\mu P(x). \end{aligned}$$
- If $0<\alpha _0< \frac{\nu ^2}{4},$ then $\mu =\frac{\nu -\sqrt{\nu ^2-4\alpha _0}}{2}$ and there exists a symmetric positive definite matrix *P*(*x*) such that
  $$\begin{aligned} Q(x)P(x)+P(x)Q^T(x)\ge 2\mu P(x). \end{aligned}$$
- If $\alpha _0 =\frac{\nu ^2}{4},$ then $\mu =\frac{\nu }{2}$ and, for any $\varepsilon \in (0, \nu ),$ there exists a symmetric positive definite matrix $P(x,\varepsilon )$ such that
  $$\begin{aligned} Q(x)P(x,\varepsilon )+P(x,\varepsilon )Q^T(x)\ge (2\mu -\varepsilon ) P(x,\varepsilon ). \end{aligned}$$

### Proof

Part 1) Let *x* be any point of $\mathbb {R}^n,$ we compute the eigenvalues $\beta (x)$ of *Q*(*x*). If $\beta (x)\ne 0$ we have the condition

$$\begin{aligned} \det (Q(x)-\beta (x) I)&=\begin{vmatrix} -\beta (x) I&I\\ -\frac{\partial ^2 V(x)}{\partial x^2}&(\nu -\beta (x)) I \end{vmatrix}\\ {}&=\frac{1}{(\beta (x))^n} \begin{vmatrix} -\beta (x) I&0\\ -\frac{\partial ^2 V(x)}{\partial x^2}&-\frac{\partial ^2 V(x)}{\partial x^2}+\beta (x)(\nu -\beta (x))I \end{vmatrix}\\ {}&={(-1)^n }\det \left( -\frac{\partial ^2 V(x)}{\partial x^2}+\beta (x)(\nu -\beta (x))I\right) \\&=0. \end{aligned}$$

Let $\alpha _i(x)\in \mathbb {R}, \, \, i \in \{1,...,n\}$ denote the eigenvalues of $\frac{\partial ^2 V(x)}{\partial x^2},$ then the above eigenvalue condition reads

$$\begin{aligned} \prod _{i=1}^n(\beta ^2(x)-\nu \beta (x)+\alpha _i(x))=0. \end{aligned}$$

Hence the non-zero eigenvalues of *Q*(*x*) are

37 $$\begin{aligned} \beta ^{\pm }_i(x)={\left\{ \begin{array}{ll} \frac{\nu \pm \sqrt{{\nu }^2-4 \alpha _i(x)}}{2}, \, \, \, \,\, \, \text {if }\, \, \, \, \nu ^2 \ge 4 \alpha _i(x) \\ \frac{\nu \pm \textbf{i} \sqrt{4 \alpha _i(x)-{\nu }^2}}{2}, \, \, \, \, \, \text {if }\, \, \, \, \nu ^2 < 4 \alpha _i(x) \end{array}\right. }, \, \, i \in \{1,...,n\}, \end{aligned}$$

where $\textbf{i}=\sqrt{-1}.$ Moreover, $\beta (x)=0$ can be an eigenvalue of *Q*(*x*) iff one of the eigenvalues of $\frac{\partial ^2 V(x)}{\partial x^2}$ is zero. This shows that *Q*(*x*) is positive stable (i.e., the eigenvalues $\beta _{i}(x)$ have positive real part) iff $\frac{\partial ^2 V(x)}{\partial x^2}> 0.$

For Part 2) we shall construct matrices *P*(*x*),  which relies on the proof of Lemma 4.3 (Lemma 4.3 in [3]).

(*a*) Let $\alpha _0> \frac{\nu ^2}{4}.$ In this case, because of (37) the matrix *Q*(*x*) is positive stable and $\mu =\frac{\nu }{2}>0.$ We define the matrix

$$\begin{aligned} P(x){:}{=}\begin{pmatrix} 2I&{}\nu I\\ \nu I&{} 2\frac{\partial ^2 V(x)}{\partial x^2} \end{pmatrix}, \end{aligned}$$

and for this choice, it is easy to check that

$$\begin{aligned} Q(x)P(x)+P(x)Q^T(x)=\nu P(x)=2\mu P(x). \end{aligned}$$

To make sure that *P*(*x*) is positive definite, we compute the eigenvalues $\eta (x)$ of *P*(*x*) at each $x \in \mathbb {R}^n:$ For $\eta (x)\ne 2$ we have the condition

$$\begin{aligned} \det (P(x)-\eta (x) I)&=\begin{vmatrix} (2-\eta (x)) I&\nu I\\ \nu I&2\frac{\partial ^2 V(x)}{\partial x^2}-\eta (x) I \end{vmatrix}\\ {}&=\frac{1}{(2-\eta (x))^n} \begin{vmatrix} (2-\eta (x)) I&0\\ \nu I&(2-\eta (x))\left( 2\frac{\partial ^2 V(x)}{\partial x^2}-\eta (x) I\right) -\nu ^2 I \end{vmatrix}\\ {}&= \det \left( (2-\eta (x))\left( 2\frac{\partial ^2 V(x)}{\partial x^2}-\eta (x) I\right) -\nu ^2 I\right) =0. \end{aligned}$$

$\eta (x)=2$ is not an eigenvalue of *P*(*x*) and so the eigenvalues of *P*(*x*) satisfy

$$\begin{aligned} \prod _{i=1}^n\left( \eta ^2(x)-(2+2\alpha _i(x))\eta (x)+4\alpha _i(x)-\nu ^2\right) =0. \end{aligned}$$

We conclude that the eigenvalues are

$$\begin{aligned} \eta ^{\pm }_i(x)=1+\alpha _i(x)\pm \sqrt{(\alpha _i(x)+1)^2-(4\alpha _i(x)-\nu ^2)}, \, \, i \in \{1,...,n\}. \end{aligned}$$

Since we assumed $\alpha _i(x)\ge \alpha (x)\ge \alpha _0> \frac{\nu ^2}{4}$ for all $i\in \{1,...,n\},$ the eigenvalues are positive and satisfy

$$\begin{aligned} \eta {:}{=}\inf _{x \in \mathbb {R}^n, \,i\in \{1,...,n\}}{\eta ^{\pm }_i(x)}=1+\alpha _0- \sqrt{(\alpha _0+1)^2-(4\alpha _0-\nu ^2)}>0. \end{aligned}$$

Thus, *P*(*x*) is positive definite and $P(x)\ge \eta I$ for all $x \in \mathbb {R}^n.$

$(b)-(c)$ Let $0<\alpha _0\le \frac{\nu ^2}{4}$. Then (37) shows $\mu =\frac{\nu -\sqrt{\nu ^2-4\alpha _0}}{2}$. Let $\varepsilon >0$ be a fixed small number. We define

$$\begin{aligned} \omega {:}{=}{\left\{ \begin{array}{ll}\alpha _0, \, \, \text { if } \alpha _0<\frac{\nu ^2}{4}\\ \alpha _0-\frac{\varepsilon ^2}{4}, \, \, \text { if } \alpha _0=\frac{\nu ^2}{4}\end{array}\right. } \end{aligned}$$

and consider the matrix

$$\begin{aligned} P(x){:}{=}\begin{pmatrix} 2I&{}\nu I\\ \nu I&{} 2\frac{\partial ^2 V(x)}{\partial x^2}+(\nu ^2-4\omega )I \end{pmatrix}. \end{aligned}$$

We compute its eigenvalues $\eta (x)$ by a similar computation as above:

38 $$\begin{aligned} \eta ^{\pm }_i(x)=1+\zeta _i(x)\pm \sqrt{(\zeta _i(x)+1)^2-(4\zeta _i(x)-\nu ^2)}, \end{aligned}$$

where $\zeta _i(x){:}{=}\alpha _i(x)+\frac{\nu ^2}{2}-2\omega >\frac{\nu ^2}{4}.$ We also have

$$\begin{aligned} \eta :=\inf _{x \in \mathbb {R}^n, \,i\in \{1,...,n\}}{\eta ^{\pm }_i(x)}={ 1+\alpha _0+\frac{\nu ^2}{2}-2\omega - \sqrt{\Big (\alpha _0+\frac{\nu ^2}{2}-2\omega -1\Big )^2+\nu ^2}} >0. \end{aligned}$$

Thus, *P*(*x*) is positive definite and $P(x)\ge \eta I$ for all $x \in \mathbb {R}^n.$ Then we compute

39 $$\begin{aligned}&Q(x)P(x)+P(x)Q^T(x)\nonumber \\&= \big (\nu -\sqrt{\nu ^2-4\omega }\big ) P(x)+\sqrt{\nu ^2-4\omega }\begin{pmatrix} 2 I&{}\big (\nu +\sqrt{\nu ^2-4\omega }\big ) I\\ \big (\nu +\sqrt{\nu ^2-4\omega }\big )I&{} 2\frac{\partial ^2 V}{\partial x^2}+\sqrt{\nu ^2-4\omega }\big (\nu +\sqrt{\nu ^2-4\omega }\big ) I \end{pmatrix}. \end{aligned}$$

Since $\frac{\partial ^2 V}{\partial x^2}\ge \omega I,$ the second matrix in the last line of (39) is bounded below by

$$\begin{aligned} \begin{pmatrix} 2 I&{}\big (\nu +\sqrt{\nu ^2-4\omega }\big ) I\\ \big (\nu +\sqrt{\nu ^2-4\omega }\big )I&{} 2\omega +\sqrt{\nu ^2-4\omega }\big (\nu +\sqrt{\nu ^2-4\omega }\big ) I \end{pmatrix}\\ =\begin{pmatrix} 2 I&{}\big (\nu +\sqrt{\nu ^2-4\omega }\big ) I\\ \big (\nu +\sqrt{\nu ^2-4\omega }\big )I&{} \frac{1}{2}\big (\nu +\sqrt{\nu ^2-4\omega }\big )^2 I \end{pmatrix}\ge 0. \end{aligned}$$

Consequently, we get

$$\begin{aligned} Q(x)P(x)+P(x)Q^T(x)\ge \big (\nu -\sqrt{\nu ^2-4\omega }\big )P(x) \, \, \, \text { for all }\, \, \, x \in \mathbb {R}^n. \end{aligned}$$

$$\square $$

Lemma 4.4 shows that, if $\frac{\partial ^2 V(x)}{\partial x^2}$ is not positive definite at some $x \in \mathbb {R}^n$ (and hence $\alpha _0\le 0$), then *Q*(*x*) is not positive stable. In this case, it is not possible to find a positive constant $\mu$ and a positive definite matrix *P*(*x*) such that $Q(x)P(x)+P(x)Q^T(x)\ge \mu P(x).$ If $\alpha _0$ is just finite and not necessarily positive, we have the following modified inequality.

### Lemma 4.5

Let $\alpha _0>-\infty .$ Then there exist $\gamma \ge 0,$ $\delta \in [0, \nu ),$ and a symmetric positive definite matrix function *P*(*x*) such that

40 $$\begin{aligned} Q(x)P(x)+P(x)Q^T(x)+\gamma D\ge (\nu -\delta )P(x), \, \, \, \, \forall x \in \mathbb {R}^d, \end{aligned}$$

where $$D=\begin{pmatrix}0&{}0\\ 0&{}\sigma I\end{pmatrix} \in \mathbb {R}^{2n \times 2n}$$ is the matrix defined in (24).

### Proof

Let $a\ge 0$ be any constant such that $a+\alpha _0>\frac{\nu ^2}{4}.$ We consider the matrix

$$\begin{aligned} P(x){:}{=}\begin{pmatrix} 2I&{}\nu I\\ \nu I&{} 2\frac{\partial ^2 V(x)}{\partial x^2}+2aI \end{pmatrix}. \end{aligned}$$

In analogy to (38) we find its eigenvalues as

$$\begin{aligned} \eta ^{\pm }_i(x)=1+\zeta _i(x)\pm \sqrt{(\zeta _i(x)+1)^2-(4\zeta _i(x)-\nu ^2)}, \end{aligned}$$

where $\zeta _i(x):=\alpha _i(x)+a\ge a+\alpha _0>\frac{\nu ^2}{4},$ and $\alpha _i(x)\in \mathbb {R}, \, \, i \in \{1,...,n\}$ denote the eigenvalues of $\frac{\partial ^2 V(x)}{\partial x^2}.$ We also have

41 $$\begin{aligned} \eta {:}{=}\inf _{x\in \mathbb {R}^n, i\in \{1,...,n\}}{\eta ^{\pm }_i(x)}= \frac{4\Big ( a+\alpha _0-\frac{\nu ^2}{4}\Big )}{ 1+ a+\alpha _0+ \sqrt{( a+\alpha _0-1)^2+\nu ^2}} >0. \end{aligned}$$

Thus, *P*(*x*) is uniformly positive definite and $P(x)\ge \eta I$ for all $x \in \mathbb {R}^n.$

Next we compute

$$\begin{aligned} QP+PQ^T+\gamma D= \nu P+\begin{pmatrix} 0&{}2a I\\ 2aI&{} (2\nu a+\gamma \sigma )I \end{pmatrix} \end{aligned}$$

42 $$\begin{aligned} =(\nu -\delta ) P+\begin{pmatrix} 2\delta I&{}(\nu \delta +2a) I\\ (\nu \delta +2a)I&{} \delta (2\frac{\partial ^2 V}{\partial x^2}+2aI)+(2\nu a+\gamma \sigma )I \end{pmatrix}, \end{aligned}$$

where $\delta \in [0, \nu )$ will be chosen later. We compute the (real) eigenvalues $\theta$ of the symmetric matrix

43 $$\begin{aligned} \begin{pmatrix} 2\delta I&{}(\nu \delta +2a) I\\ (\nu \delta +2a)I&{} \delta (2\frac{\partial ^2 V}{\partial x^2}+2aI)+(2\nu a+\gamma \sigma )I \end{pmatrix} \end{aligned}$$

which appears in (42):

For $\theta (x)\ne 2 \delta$ we have the condition

$$\begin{aligned}&\begin{vmatrix} (2\delta -\theta ) I&(\nu \delta +2a) I\\ (\nu \delta +2a)I&\delta (2\frac{\partial ^2 V}{\partial x^2}+2aI)+(2\nu a+\gamma \sigma -\theta )I \end{vmatrix}\\&\quad = \frac{1}{(2\delta -\theta )^n}\begin{vmatrix} (2\delta -\theta ) I&0 \\ (\nu \delta +2a)I&(2\delta -\theta ) \left( \delta (2\frac{\partial ^2 V}{\partial x^2}+2aI)+(2\nu a+\gamma \sigma -\theta )I \right) -(\nu \delta +2a)^2 I \end{vmatrix}\\&\quad {=\bigg | (2\delta -\theta ) \left( \delta (2\frac{\partial ^2 V}{\partial x^2}+2aI)+(2\nu a+\gamma \sigma -\theta )I \right) -(\nu \delta +2a)^2 I \bigg | } \\&\quad =\prod _{i=1}^n \left( \theta ^2-\theta \left[ 2\delta (\alpha _i(x)+a)+2\delta +2\nu a+\gamma \sigma \right] +4\delta ^2(\alpha _i(x)+a-{\nu ^2}/{4})+2\delta \gamma \sigma -4a^2 \right) =0. \end{aligned}$$

Let us consider the following equations with $i \in \{1,...,n\}:$

44 $$\begin{aligned} \theta ^2{-}\theta [2\delta (\alpha _i(x){+}a){+}2\delta {+}2\nu a+\gamma \sigma ]{+}[4\delta ^2(\alpha _i(x){+}a-{\nu ^2}/{4})+2\delta \gamma \sigma -4a^2]=0,\nonumber \\ \end{aligned}$$

and we shall show that they have non-negative solutions for an appropriate choice of $\delta$ and $\gamma .$ To this end we see first that

$$\begin{aligned} 2\delta (\alpha _i(x)+a)+2\delta +2\nu a+\gamma \sigma \ge 2\delta (\alpha _0+a)+2\delta \ge \frac{\delta \nu ^2}{2}+2\delta \ge 0. \end{aligned}$$

Next, we choose

45 $$\begin{aligned} \displaystyle \delta =\delta (a,\gamma ){:}{=}\frac{1}{\sqrt{a+\alpha _0-\frac{\nu ^2}{4}}}\left[ \sqrt{\left( \frac{\gamma \sigma }{4\sqrt{a+\alpha _0-\frac{\nu ^2}{4}}}\right) ^2+a^2}-\frac{\gamma \sigma }{4\sqrt{a+\alpha _0-\frac{\nu ^2}{4}}}\right] \ge 0,\nonumber \\ \end{aligned}$$

which satisfies

46 $$\begin{aligned} 4\delta ^2\Big (a+\alpha _0-\frac{\nu ^2}{4}\Big ) +2\delta \gamma \sigma -4a^2=0. \end{aligned}$$

Hence, the last term of (44) satisfies

$$\begin{aligned} 4\delta ^2\Big (\alpha _i(x)+a-\frac{\nu ^2}{4}\Big )+2\delta \gamma \sigma -4a^2\ge 4\delta ^2\Big (a+\alpha _0-\frac{\nu ^2}{4}\Big ) +2\delta \gamma \sigma -4a^2=0 \end{aligned}$$

for all $i \in \{1,...,n\}.$ Therefore, the quadratic equations (44) have non-negative coefficients and so their solutions, i.e. the eigenvalues of (43), are non-negative. Consequently, we get (40).

We note that $\delta$ from (45) satisfies, for any fixed $a>\frac{\nu }{4}-\alpha _0,$ $\delta (a, \gamma ) \rightarrow 0$ as $\gamma \rightarrow \infty .$ Hence, choosing $\gamma$ large enough, we have $\delta \in [0,\nu ).$ $\square$

### Remark 4.6

If $\alpha _0>0,$ we can take $\gamma =0$ in Lemma 4.5. This follows by choosing in the proof of Lemma 4.5

$$\begin{aligned} a={\left\{ \begin{array}{ll} 0,&{} \text { if } \alpha _0>\frac{\nu ^2}{4}\\ \frac{\varepsilon ^2}{2},&{} \text { if } \alpha _0=\frac{\nu ^2}{4} \\ \frac{\nu ^2-4\alpha _0}{2},&{} \text { if } 0<\alpha _0<\frac{\nu ^2}{4} \end{array}\right. }, \, \, \, \, \delta ={\left\{ \begin{array}{ll} 0,&{} \text { if } \alpha _0>\frac{\nu ^2}{4}\\ \frac{\varepsilon }{\sqrt{2}},&{} \text { if } \alpha _0=\frac{\nu ^2}{4} \\ \sqrt{\nu ^2-4\alpha _0},&{} \text { if } 0<\alpha _0<\frac{\nu ^2}{4} \end{array}\right. }, \end{aligned}$$

with any $\varepsilon \in (0, \nu ).$ Therefore, Lemma 4.5 includes the second part of Lemma 4.4. However, if $\alpha _0\le 0,$ we have to choose $\gamma >0.$

## Proofs

### Proof of Theorem 2.3

#### Proof

We denote $\displaystyle u_1{:}{=}\nabla _{x}\left( \frac{f}{f_{\infty }}\right) ,\, \, u_2{:}{=}\nabla _{v}\left( \frac{f}{f_{\infty }}\right) ,$ and $$ u{:}{=}\begin{pmatrix} u_1\\ u_2 \end{pmatrix}.$$

We consider the modified dissipation functional

$$\begin{aligned} S(f(t))=2 \int _{\mathbb {R}^{2n}}u^T(t) Pu(t) f_{\infty }dxdv \end{aligned}$$

for some symmetric positive definite matrix $P=P(x,v)\in \mathbb {R}^{2n \times 2n}.$ By Lemma 4.1 (for a *t*-independent matrix *P*) we have

47 $$\begin{aligned} \displaystyle \frac{d}{dt} S(f(t))&=-4\sigma \int _{\mathbb {R}^{2n}}\left\{ \sum _{i=1}^n (\partial _{v_i}u)^TP\partial _{v_i}u\right\} f_{\infty }dxdv\nonumber \\&\quad -2\int _{\mathbb {R}^{2n}} u^T\left\{ QP+PQ^T\right\} u f_{\infty }dxdv\nonumber \\&\quad -2\int _{\mathbb {R}^{2n}} u^T\left\{ [\nabla _x V\cdot \nabla _v-v\cdot \nabla _x+\nu v\cdot \nabla _v-\sigma \Delta _v]P\right\} u f_{\infty }dxdv, \end{aligned}$$

with $$Q(x)= \begin{pmatrix} 0&{}I\\ -\frac{\partial ^2 V(x)}{\partial x^2}&{}\nu I \end{pmatrix}.$$ Let $c\in \mathbb {R}$ and $\tau \in [0,\nu )$ are the constants such that Assumption 2.2 is satisfied. Since (6) is positive semi-definite, $\frac{\partial ^2 V(x)}{\partial x^2}+cI$ is also positive semi-definite and so $\frac{\partial ^2 V(x)}{\partial x^2}\ge -cI$ for all $x \in \mathbb {R}^n$. We define the matrix *P* depending on the constant *c*.

**Case** (*a*) :  Assume $c\le -\frac{\nu ^2}{4}, \,\, \alpha _0>\frac{\nu ^2}{4}.$ By Lemma 4.4 (2*a*) and by its proof, the matrix $$P(x){:}{=}\begin{pmatrix} 2I&{}\nu I\\ \nu I&{} 2\frac{\partial ^2 V(x)}{\partial x^2} \end{pmatrix}$$ satisfies

$$\begin{aligned} Q(x)P(x)+P(x)Q^T(x)=\nu P(x)\, \,\text { and } \, \,P(x)\ge \eta I \end{aligned}$$

for all $x \in \mathbb {R}^n$ and $\eta {:}{=}1+\alpha _0- \sqrt{(\alpha _0+1)^2-(4\alpha _0-\nu ^2)}>0.$ For this choice of the matrix *P*, 

48 $$\begin{aligned}{}[\nabla _x V\cdot \nabla _v-v\cdot \nabla _x+\nu v\cdot \nabla _v-\sigma \Delta _v]P(x)=\begin{pmatrix} 0&{}0\\ 0&{} -2\frac{\partial ^2 (v\cdot \nabla _x V)}{\partial x^2} \end{pmatrix}. \end{aligned}$$

Then (47) can be written as

49 $$\begin{aligned} \frac{d}{dt} S(f(t))&=-4\sigma \int _{\mathbb {R}^{2n}}\left\{ \sum _{i=1}^n (\partial _{v_i}u)^TP\partial _{v_i}u\right\} f_{\infty }dxdv\nonumber \\&\quad -2\nu \int _{\mathbb {R}^{2n}} u^T Pu f_{\infty }dxdv +4\int _{\mathbb {R}^{2n}} u^T\begin{pmatrix} 0&{}0\\ 0&{} \frac{\partial ^2 (v\cdot \nabla _x V)}{\partial x^2} \end{pmatrix} u f_{\infty }dxdv\nonumber \\&= -4\sigma \int _{\mathbb {R}^{2n}}\left\{ \sum _{i=1}^n (\partial _{v_i}u)^TP\partial _{v_i}u\right\} f_{\infty }dxdv-\nu S(f(t))\nonumber \\&\quad +4\int _{\mathbb {R}^{2n}} u^T\begin{pmatrix} 0&{}0\\ 0&{} \frac{\partial ^2 (v\cdot \nabla _x V)}{\partial x^2} \end{pmatrix} u f_{\infty }dxdv. \end{aligned}$$

We shall now consider each term of this equation. First we compute

50 $$\begin{aligned}&S(f(t))=2\int _{\mathbb {R}^{2n}}\left\{ 2 |u_{1}|^2+2\nu u_{1}\cdot u_{2}+2u^T_{2}\frac{\partial ^2 V}{\partial x^2}u_{2}\right\} f_{\infty }dxdv \nonumber \\&\quad =4\int _{\mathbb {R}^{2n}}|u_{1}+\frac{\nu }{2} u_{2}|^2f_{\infty }dxdv+4\int _{\mathbb {R}^{2n}} u^T_{2}\left( \frac{\partial ^2 V}{\partial x^2}-\frac{\nu ^2}{4}I\right) u_{2} f_{\infty }dxdv \nonumber \\&\quad \ge 4\int _{\mathbb {R}^{2n}} u^T_{2}\left( \frac{\partial ^2 V}{\partial x^2}-\frac{\nu ^2}{4}I\right) u_{2} f_{\infty }dxdv. \end{aligned}$$

Then

51 $$\begin{aligned}&\displaystyle 4\sigma \int _{\mathbb {R}^{2n}}\left\{ \sum _{i=1}^n (\partial _{v_i}u)^TP \partial _{v_i}u\right\} f_{\infty }dxdv \nonumber \\&\quad =4\sigma \int _{\mathbb {R}^{2n}}\left\{ \sum _{i=1}^n\left( 2 |\partial _{v_i}u_1|^2+2\nu \partial _{v_i}u_1\cdot \partial _{v_i}u_2+2(\partial _{v_i}u_2)^T\frac{\partial ^2 V}{\partial x^2}\partial _{v_i}u_2\right) \right\} f_{\infty }dxdv\nonumber \\&\quad = 8\sigma \int _{\mathbb {R}^{2n}}\left\{ \sum _{i=1}^n |\partial _{v_i}u_1+\frac{\nu }{2} \partial _{v_i}u_2|^2\right\} f_{\infty }dxdv\nonumber \\&\qquad +8\sigma \int _{\mathbb {R}^{2n}}\left\{ \sum _{i=1}^n (\partial _{v_i}u_2)^T\left( \frac{\partial ^2 V}{\partial x^2}-\frac{\nu ^2}{4}I\right) \partial _{v_i}u_2\right\} f_{\infty }dxdv \nonumber \\&\quad \ge 8\sigma \int _{\mathbb {R}^{2n}}\left\{ \sum _{i=1}^n (\partial _{v_i}u_2)^T\left( \frac{\partial ^2 V}{\partial x^2}-\frac{\nu ^2}{4}I\right) \partial _{v_i}u_2\right\} f_{\infty }dxdv. \end{aligned}$$

Now we consider the last term in (49)

52 $$\begin{aligned}&\displaystyle 4\int _{\mathbb {R}^{2n}} u^T\begin{pmatrix} 0&{}0\\ 0&{} \frac{\partial ^2 (v\cdot \nabla _x V)}{\partial x^2} \end{pmatrix} u f_{\infty }dxdv=4\int _{\mathbb {R}^{2n}} u_2^T\frac{\partial ^2 (v\cdot \nabla _x V)}{\partial x^2} u_2 f_{\infty }dxdv\nonumber \\&\quad = 4\int _{\mathbb {R}^{2n}}\left\{ \sum _{i,j=1}^n u_{2,i} v\cdot \nabla _x V_{ij}u_{2,j}\right\} f_{\infty }dxdv=4\int _{\mathbb {R}^{2n}}\left\{ \sum _{i,j,k=1}^n u_{2,i} v_k V_{ijk}u_{2,j}\right\} f_{\infty }dxdv\nonumber \\&\quad =-\frac{4\sigma }{\nu }\int _{\mathbb {R}^{2n}}\left\{ \sum _{i,j,k=1}^n u_{2,i} V_{ijk}u_{2,j}(\partial _{v_k}f_{\infty })\right\} dxdv\nonumber \\&\quad =\frac{4\sigma }{\nu }\int _{\mathbb {R}^{2n}}\left\{ \sum _{i,j,k=1}^n \partial _{v_k}(u_{2,i} u_{2,j}) V_{ijk}\right\} f_{\infty }dxdv\nonumber \\&\quad = \frac{4\sigma }{\nu }\int _{\mathbb {R}^{2n}}\left\{ \sum _{i,j,k=1}^n (\partial _{v_k}u_{2,i}) u_{2,j} V_{ijk}+u_{2,i}(\partial _{v_k}u_{2,j}) V_{ijk}\right\} f_{\infty }dxdv\nonumber \\&\quad =\frac{8\sigma }{\nu }\int _{\mathbb {R}^{2n}}\left\{ \sum _{i,j,k=1}^n (\partial _{v_k}u_{2,i}) u_{2,j} V_{ijk}\right\} f_{\infty }dxdv\nonumber \\&\quad =\frac{8\sigma }{\nu }\int _{\mathbb {R}^{2n}}\left\{ \sum _{k=1}^n (\partial _{v_k} u_2)^T \frac{\partial ^2(\partial _{x_k}V)}{\partial x^2} u_{2} \right\} f_{\infty }dxdv, \end{aligned}$$

where we integrated by parts and used $\partial _{v_k} f_{\infty }=-\frac{\nu }{\sigma }v_k f_{\infty }$ and the notations $u_{2,i}{:}{=}\partial _{v_i}\left( \frac{f}{f_{\infty }}\right) ,$ $V_{ij}{:}{=}\partial _{x_ix_j}^2V,$ $V_{ijk}{:}{=}\partial ^3_{x_ix_jx_k}V.$ By (49), (51), (52), and (50) we obtain

$$\begin{aligned}&\displaystyle \frac{d}{dt} S(f(t))+(\nu -\tau )S(f(t))\le -\tau S(f(t))\\&\qquad -8\sigma \int _{\mathbb {R}^{2n}}\left\{ \sum _{i=1}^n (\partial _{v_i}u_2)^T\left( \frac{\partial ^2 V}{\partial x^2}-\frac{\nu ^2}{4}I\right) \partial _{v_i}u_2\right\} f_{\infty }dxdv\\&\qquad + \frac{8\sigma }{\nu }\int _{\mathbb {R}^{2n}}\left\{ \sum _{i=1}^n (\partial _{v_i}u_{2})^T \frac{\partial ^2(\partial _{x_i}V)}{\partial x^2} u_{2} \right\} f_{\infty }dxdv\\&\quad \le -4\tau \int _{\mathbb {R}^{2n}} u^T_{2}\left( \frac{\partial ^2 V}{\partial x^2}-\frac{\nu ^2}{4}I\right) u_{2}f_{\infty }dxdv\\&\qquad -8\sigma \int _{\mathbb {R}^{2n}}\left\{ \sum _{i=1}^n (\partial _{v_i}u_2)^T\left( \frac{\partial ^2 V}{\partial x^2}-\frac{\nu ^2}{4}I\right) \partial _{v_i}u_2\right\} f_{\infty }dxdv\\&\qquad +\frac{8\sigma }{\nu }\int _{\mathbb {R}^{2n}}\left\{ \sum _{i=1}^n (\partial _{v_i}u_2)^T \frac{\partial ^2(\partial _{x_i}V)}{\partial x^2} u_{2} \right\} f_{\infty }dxdv \\&\quad {=}{-}\frac{8\sigma }{\nu }\sum _{i{=}1}^n\int _{\mathbb {R}^{2n}} \left\{ \nu (\partial _{v_i}u_2)^T\left( \frac{\partial ^2 V}{\partial x^2}{-}\frac{\nu ^2}{4}I\right) \partial _{v_i}u_2{-}(\partial _{v_i}u_2)^T \frac{\partial ^2(\partial _{x_i}V)}{\partial x^2} u_{2}\right\} f_{\infty }dxdv\\&\qquad -\frac{8\sigma }{\nu }\int _{\mathbb {R}^{2n}}\frac{\tau \nu }{2\sigma }u^T_{2}\left( \frac{\partial ^2 V}{\partial x^2}-\frac{\nu ^2}{4}I\right) u_{2}f_{\infty }dxdv. \end{aligned}$$

The right hand side of this inequality is a quadratic polynomial with respect to $\partial _{v_i}u_{2}, \, \, i\in \{1,...,n\},$ and $u_2.$ The corresponding matrix of this quadratic polynomial is

53 $$\begin{aligned} \begin{pmatrix} \nu \left( \frac{\partial ^2 V}{\partial x^2}-\frac{\nu ^2}{4}I\right) &{}0&{}...&{}0&{}-\frac{1}{2}\frac{\partial ^2(\partial _{x_1}V)}{\partial x^2} \\ 0&{}\nu \left( \frac{\partial ^2 V}{\partial x^2}-\frac{\nu ^2}{4}I\right) &{}...&{}0&{}-\frac{1}{2}\frac{\partial ^2(\partial _{x_2}V)}{\partial x^2}\\ ...&{}...&{}...&{}...&{}...\\ 0&{}0&{}...&{}\nu \left( \frac{\partial ^2 V}{\partial x^2} -\frac{\nu ^2}{4}I\right) &{}-\frac{1}{2}\frac{\partial ^2(\partial _{x_n}V)}{\partial x^2}\\ -\frac{1}{2}\frac{\partial ^2(\partial _{x_1}V)}{\partial x^2}&{}-\frac{1}{2}\frac{\partial ^2(\partial _{x_2}V)}{\partial x^2}&{}...&{}-\frac{1}{2}\frac{\partial ^2(\partial _{x_n}V)}{\partial x^2}&{}\frac{\tau \nu }{2\sigma }\left( \frac{\partial ^2 V}{\partial x^2}-\frac{\nu ^2}{4}I\right) \end{pmatrix}. \end{aligned}$$

The assumption $\frac{\partial ^2 V}{\partial x^2}-\frac{\nu ^2}{4}I\ge \frac{\partial ^2 V}{\partial x^2}+cI$ and the Assumption 2.2 imply that (53) is positive semi-definite.

Thus we have obtained

$$\begin{aligned} \displaystyle \frac{d}{dt} S(f(t))+(\nu -\tau )S(f(t))\le 0 \end{aligned}$$

and by Grönwall’s lemma

54 $$\begin{aligned} S(f(t))\le e^{-(\nu -\tau )t}S(f_0). \end{aligned}$$

The estimate $P(x)\ge \eta I$ and the Poincaré inequality (5) imply

55 $$\begin{aligned} \int _{\mathbb {R}^{2n}}\left( \frac{f(t)}{f_{\infty }}-1\right) ^2 f_{\infty }dxdv\le \frac{1}{2C_{PI}\eta } S(f(t))\le \frac{1}{2C_{PI}\eta } e^{-(\nu -\tau )t}S(f_0). \end{aligned}$$

The matrix inequalities (see Lemma 6.1 in Appendix 6.2)

56 $$\begin{aligned}{} & {} \frac{1}{1+\alpha _0+\sqrt{(1-\alpha _0)^2+\nu ^2}}P \le \begin{pmatrix} I&{}0\\ 0&{} \frac{\partial ^2 V}{\partial x^2}+(1-\alpha _0)I \end{pmatrix}\nonumber \\{} & {} \quad \le \frac{1+\alpha _0+\sqrt{(1-\alpha _0)^2+\nu ^2}}{4\alpha _0-\nu ^2}P \end{aligned}$$

show that *S*(*f*(*t*)) is equivalent to the functional

$$\begin{aligned} \int _{\mathbb {R}^{2n}}\left| \nabla _{x}\left( \frac{f(t)}{f_{\infty }}\right) \right| ^2 f_{\infty }dxdv{+}\int _{\mathbb {R}^{2n}}\nabla ^T_{v}\left( \frac{f(t)}{f_{\infty }}\right) \left( \frac{\partial ^2 V}{\partial x^2}{+} (1{-}\alpha _0) I\right) \nabla _{v}\left( \frac{f(t)}{f_{\infty }}\right) f_{\infty } dxdv. \end{aligned}$$

This equivalence, and (55) let us obtain (9).

 **Case** (*b*) :  Assume $$c=-\alpha _0=-\frac{\nu ^2}{4}.$$

Then by Lemma 4.4 (2*c*),  for any $\varepsilon \in (0,\nu -\tau ),$ the matrix

$$\begin{aligned} P(x){:}{=}\begin{pmatrix} 2I&{}\nu I\\ \nu I&{} 2\frac{\partial ^2 V(x)}{\partial x^2}+\varepsilon ^2 I \end{pmatrix} \end{aligned}$$

satisfies

57 $$\begin{aligned} Q(x)P(x)+P(x)Q^T(x)\ge (\nu -{\varepsilon })P(x) \, \, \, \, \text {and} \, \, \, P(x)\ge \eta I \end{aligned}$$

for all $x \in \mathbb {R}^n$ and $\eta {:}{=}{ 1+\frac{\nu ^2+2\varepsilon ^2}{4}- \sqrt{(\frac{\nu ^2+2\varepsilon ^2}{4}-1)^2+\nu ^2}} >0.$ With this matrix we have

58 $$\begin{aligned}&S(f(t))=4\int _{\mathbb {R}^{2n}}|u_{1}+\frac{\nu }{2} u_{2}|^2f_{\infty }dxdv+4\int _{\mathbb {R}^{2n}} u^T_{2}\left( \frac{\partial ^2 V}{\partial x^2}+\frac{2\varepsilon ^2-\nu ^2}{4}I\right) u_{2} f_{\infty }dxdv\nonumber \\&\quad \ge 4\int _{\mathbb {R}^{2n}} u^T_{2}\left( \frac{\partial ^2 V}{\partial x^2}+\frac{2\varepsilon ^2-\nu ^2}{4}I\right) u_{2} f_{\infty }dxdv, \end{aligned}$$

59 $$\begin{aligned}&4\sigma \int _{\mathbb {R}^{2n}}\left\{ \sum _{i=1}^n (\partial _{v_i}u)^T P \partial _{v_i}u\right\} f_{\infty }dxdv\nonumber \\&\quad = 8\sigma \int _{\mathbb {R}^{2n}}\left\{ \sum _{i=1}^n |\partial _{v_i}u_{1}+\frac{\nu }{2}\partial _{v_i} u_{2}|^2\right\} f_{\infty }dxdv \nonumber \\&\qquad +8\sigma \int _{\mathbb {R}^{2n}}\left\{ \sum _{i=1}^n (\partial _{v_i}u_2)^T\left( \frac{\partial ^2 V}{\partial x^2}+\frac{2\varepsilon ^2-\nu ^2}{4}I\right) \partial _{v_i}u_2\right\} f_{\infty }dxdv, \end{aligned}$$

and by using (48), $\partial _{v_i}f_{\infty }=-\frac{\nu }{\sigma }v_i f_{\infty }:$

60 $$\begin{aligned}&-2\int _{\mathbb {R}^{2n}} u^T\left\{ [\nabla _x V\cdot \nabla _v-v\cdot \nabla _x+\nu v\cdot \nabla _v-\sigma \Delta _v]P\right\} u f_{\infty }dxdv \nonumber \\&\quad =\frac{8\sigma }{\nu }\int _{\mathbb {R}^{2n}}\left\{ \sum _{i=1}^n(\partial _{v_i}u_2)^T \frac{\partial ^2(\partial _{x_i}V)}{\partial x^2} u_{2} \right\} f_{\infty }dxdv. \end{aligned}$$

(47), (57), (58), (59), (60), and similar estimates as for Case *a*) show that

$$\begin{aligned}&\displaystyle \frac{d}{dt} S(f(t))+(\nu -\tau -\varepsilon )S(f(t)) \\&\quad \le {-}\frac{8\sigma }{\nu }\sum _{i{=}1}^n\int _{\mathbb {R}^{2n}}\left\{ \nu (\partial _{v_i}u_2)^T\left( \frac{\partial ^2 V}{\partial x^2}{+}\frac{2\varepsilon ^2{-}\nu ^2}{4}I\right) \partial _{v_i}u_{2}{-}(\partial _{v_i}u_2)^T \frac{\partial ^2(\partial _{x_i}V)}{\partial x^2} u_{2}\right\} f_{\infty }dxdv \\&\qquad -\frac{8\sigma }{\nu }\int _{\mathbb {R}^{2n}}\frac{\tau \nu }{2\sigma }u^T_{2}\left( \frac{\partial ^2 V}{\partial x^2}+\frac{2\varepsilon ^2-\nu ^2}{4}I\right) u_{2}f_{\infty }dxdv. \end{aligned}$$

The right hand side of this inequality is a quadratic polynomial with respect to $\partial _{v_i}u_{2}, \, \, i\in \{1,...,n\},$ and $u_2.$ The corresponding matrix of this quadratic polynomial is

61 $$\begin{aligned} \begin{pmatrix} \nu \left( \frac{\partial ^2 V}{\partial x^2}{+}\frac{2\varepsilon ^2{-}\nu ^2}{4}I\right) &{}0&{}...&{}0&{}{-}\frac{1}{2}\frac{\partial ^2(\partial _{x_1}V)}{\partial x^2} \\ 0&{}\nu \left( \frac{\partial ^2 V}{\partial x^2}{+}\frac{2\varepsilon ^2{-}\nu ^2}{4}I\right) &{}...&{}0&{}{-}\frac{1}{2}\frac{\partial ^2(\partial _{x_2}V)}{\partial x^2}\\ ...&{}...&{}...&{}...&{}...\\ 0&{}0&{}...&{}\nu \left( \frac{\partial ^2 V}{\partial x^2}+\frac{2\varepsilon ^2-\nu ^2}{4}I\right) &{} {-}\frac{1}{2}\frac{\partial ^2(\partial _{x_n}V)}{\partial x^2}\\ {-}\frac{1}{2}\frac{\partial ^2(\partial _{x_1}V)}{\partial x^2}&{}-\frac{1}{2}\frac{\partial ^2(\partial _{x_2}V)}{\partial x^2}&{}...&{}-\frac{1}{2}\frac{\partial ^2(\partial _{x_n}V)}{\partial x^2}&{}\frac{\tau \nu }{2\sigma }\left( \frac{\partial ^2 V}{\partial x^2}+\frac{2\varepsilon ^2-\nu ^2}{4}I\right) \end{pmatrix}.\nonumber \\ \end{aligned}$$

Because of $\frac{\partial ^2 V}{\partial x^2}+\frac{2\varepsilon ^2-\nu ^2}{4}I> \frac{\partial ^2 V}{\partial x^2}+cI$ and Assumption 2.2, (61) is positive definite and we get

$$\begin{aligned} \displaystyle \frac{d}{dt} S(f(t))+(\nu -\tau -\varepsilon )S(f(t))\le 0 \end{aligned}$$

and by Grönwall’s lemma

62 $$\begin{aligned} S(f(t))\le e^{-(\nu -\tau -\varepsilon )t}S(f_0). \end{aligned}$$

Similar to (55), we have

63 $$\begin{aligned} \int _{\mathbb {R}^{2n}}\left( \frac{f(t)}{f_{\infty }}-1\right) ^2 f_{\infty }dxdv\le \frac{1}{2C_{PI}\eta } S(f(t))\le \frac{1}{2C_{PI}\eta } e^{-(\nu -\tau -\varepsilon )t}S(f_0). \end{aligned}$$

The functional

$$\begin{aligned} \int _{\mathbb {R}^{2n}}\left| \nabla _{x}\left( \frac{f(t)}{f_{\infty }}\right) \right| ^2 f_{\infty }dxdv{+}\int _{\mathbb {R}^{2n}}\nabla ^T_{v}\left( \frac{f(t)}{f_{\infty }}\right) \left( \frac{\partial ^2 V}{\partial x^2}{+} (1{-}\alpha _0) I\right) \nabla _{v}\left( \frac{f(t)}{f_{\infty }}\right) f_{\infty } dxdv \end{aligned}$$

and *S*(*f*(*t*)) are equivalent because of (see Lemma 6.1 in Appendix 6.2)

64 $$\begin{aligned}{} & {} \frac{1}{1+\frac{\nu ^2+2\varepsilon ^2}{4}+\sqrt{\left( 1-\frac{\nu ^2+2\varepsilon ^2}{4}\right) ^2+\nu ^2}}P \le \begin{pmatrix} I&{}0\\ 0&{} \frac{\partial ^2 V}{\partial x^2}+(1-\alpha _0)I \end{pmatrix}\nonumber \\{} & {} \quad \le \frac{1+\frac{\nu ^2+2\varepsilon ^2}{4} +\sqrt{\left( 1-\frac{\nu ^2+2\varepsilon ^2}{4}\right) ^2+\nu ^2}}{2\varepsilon ^2}P. \end{aligned}$$

This equivalence, and (63) imply (9).

 **Case** (*c*) **and** (*d*),  **exponential decay**: Assume $c>-\frac{\nu ^2}{4}.$ For some $\gamma \ge 0$ to be chosen later, we consider the functional

65 $$\begin{aligned} \Phi (f(t)) {:}{=}&\gamma \int _{\mathbb {R}^{2n}}\left( \frac{f}{f_{\infty }}-1\right) ^2 f_{\infty }d xdv+S(f(t))\nonumber \\=&\gamma \int _{\mathbb {R}^{2n}}\left( \frac{f}{f_{\infty }}-1\right) ^2 f_{\infty }dxdv+2\int _{\mathbb {R}^{2n}}u^TPuf_{\infty }dxdv. \end{aligned}$$

Using (17) and (47) its time derivative reads

$$\begin{aligned} \frac{d \Phi (f(t))}{dt}{} & {} =-4\sigma \int _{\mathbb {R}^{2n}}\left\{ \sum _{i=1}^n (\partial _{v_i}u)^TP\partial _{v_i} u\right\} f_{\infty }dxdv\\{} & {} \quad -2\int _{\mathbb {R}^{2n}} u^T\left\{ QP+PQ^T+\gamma D\right\} u f_{\infty }dxdv \end{aligned}$$

66 $$\begin{aligned} -2\int _{\mathbb {R}^{2n}} u^T\left\{ [\nabla _x V\cdot \nabla _v-v\cdot \nabla _x+\nu v\cdot \nabla _v-\sigma \Delta _v]P\right\} u f_{\infty }dxdv. \end{aligned}$$

Let *a*,  to be chosen later, be any number such that $a\ge c+\frac{\nu ^2}{4}>0$ and $a+\alpha _0>\frac{\nu ^2}{4}.$ We consider the matrix

67 $$\begin{aligned} P(x){:}{=}\begin{pmatrix} 2I&{}\nu I\\ \nu I&{} 2\frac{\partial ^2 V(x)}{\partial x^2}+2aI \end{pmatrix}. \end{aligned}$$

Then, by Lemma 4.5 we have

68 $$\begin{aligned} Q(x)P(x)+P(x)Q^T(x)+\gamma D\ge (\nu -\delta )P(x), \, \, \, \, \, \forall x \in \mathbb {R}^d, \end{aligned}$$

with a constant $\delta$ defined in (45). If $\gamma$ is large enough, (45) shows that $\delta \in (0,\nu -\tau ).$

The choice of the matrix *P* in (67), (66), and (68) lets us estimate

69 $$\begin{aligned}&\frac{d \Phi (f(t))}{dt}\le -4\sigma \int _{\mathbb {R}^{2n}}\left\{ \sum _{i=1}^n(\partial _{v_i}u)^T P \partial _{v_i}u \right\} f_{\infty }dxdv\nonumber \\&-(\nu -\delta )S(f(t))+4\int _{\mathbb {R}^{2n}} u^T\begin{pmatrix} 0&{}0\\ 0&{} \frac{\partial ^2 (v\cdot \nabla _x V)}{\partial x^2} \end{pmatrix} u f_{\infty }dxdv. \end{aligned}$$

Similar computations as for Case (*a*) as well as (58) (but with $\varepsilon ^2=2a$) lead to

$$\begin{aligned}&\displaystyle \frac{d}{dt} \Phi (f(t))+(\nu -\delta -\tau )S(f(t))\\&\quad \le -\frac{8\sigma }{\nu }\sum _{i=1}^n\int _{\mathbb {R}^{2n}}\left\{ \nu (\partial _{v_i}u_2)^T\left( \frac{\partial ^2 V}{\partial x^2}+\frac{4a-\nu ^2}{4}I\right) \partial _{v_i}u_{2}-(\partial _{v_i}u_{2})^T \frac{\partial ^2(\partial _{x_i}V)}{\partial x^2} u_{2}\right\} f_{\infty }dxdv\\&\qquad -\frac{8\sigma }{\nu }\int _{\mathbb {R}^{2n}}\frac{\tau \nu }{2\sigma }u^T_{2}\left( \frac{\partial ^2 V}{\partial x^2}+\frac{4a-\nu ^2}{4}I\right) u_{2}f_{\infty }dxdv. \end{aligned}$$

The two integrands of the right hand side are together a quadratic polynomial of $\partial _{v_i}u_{2},$ $i\in \{1,...,n\},$ and $u_2,$ and its corresponding matrix is

70 $$\begin{aligned} \begin{pmatrix} \nu \left( \frac{\partial ^2 V}{\partial x^2}+\frac{4a-\nu ^2}{4}I\right) &{}0&{}...&{}0&{}-\frac{1}{2}\frac{\partial ^2(\partial _{x_1}V)}{\partial x^2} \\ 0&{}\nu \left( \frac{\partial ^2 V}{\partial x^2}+\frac{4a-\nu ^2}{4}I\right) &{}...&{}0&{}-\frac{1}{2}\frac{\partial ^2(\partial _{x_2}V)}{\partial x^2}\\ ...&{}...&{}...&{}...&{}...\\ 0&{}0&{}...&{}\nu \left( \frac{\partial ^2 V}{\partial x^2}+\frac{4a-\nu ^2}{4}I\right) &{}-\frac{1}{2}\frac{\partial ^2(\partial _{x_n}V)}{\partial x^2}\\ -\frac{1}{2}\frac{\partial ^2(\partial _{x_1}V)}{\partial x^2}&{}-\frac{1}{2}\frac{\partial ^2(\partial _{x_2}V)}{\partial x^2}&{}...&{}-\frac{1}{2}\frac{\partial ^2(\partial _{x_n}V)}{\partial x^2}&{}\frac{\tau \nu }{2\sigma }\left( \frac{\partial ^2 V}{\partial x^2}+\frac{4a-\nu ^2}{4}I\right) \end{pmatrix}.\nonumber \\ \end{aligned}$$

Because of $a-\frac{\nu ^2}{4}\ge c$ and Assumption 2.2, the matrix (70) is positive semi-definite, thus, we have

71 $$\begin{aligned} \displaystyle \frac{d}{dt} \Phi (f(t))+(\nu -\tau -\delta )S(f(t))\le 0. \end{aligned}$$

The estimate $P(x)\ge \eta I$ ($\eta >0$ defined in (41)) and the Poincaré inequality (5) imply

$$\begin{aligned} \int _{\mathbb {R}^{2n}}\left( \frac{f}{f_{\infty }}-1\right) ^2 f_{\infty }dxdv\le \frac{1}{ 2\eta C_{PI}} S(f(t)) \end{aligned}$$

and so

$$\begin{aligned} \frac{1}{1+\frac{\gamma }{2\eta C_{PI}}}\Phi (f(t))\le S(f(t)). \end{aligned}$$

This estimate and (71) let us conclude

72 $$\begin{aligned} \frac{d}{dt} \Phi (f(t))+2\lambda \Phi (f(t))\le 0 \end{aligned}$$

for

73 $$\begin{aligned} 2\lambda =\frac{\nu -\tau -\delta }{1+\frac{\gamma }{2\eta C_{PI}}}>0. \end{aligned}$$

By Grönwall’s lemma we obtain

74 $$\begin{aligned} \Phi (f(t))\le e^{-2\lambda t} \Phi (f_0). \end{aligned}$$

One can check that (see Lemma 6.1 in Appendix 6.2)

75 $$\begin{aligned}{} & {} \frac{1}{a+\alpha _0+1+\sqrt{(a+\alpha _0-1)^2+\nu ^2}} P \nonumber \\{} & {} \le \begin{pmatrix} I&{}0\\ 0&{} \frac{\partial ^2 V}{\partial x^2}+(1-\alpha _0)I \end{pmatrix}\le \frac{a+\alpha _0+1+\sqrt{(a+\alpha _0-1)^2+\nu ^2}}{4(a+\alpha _0)-\nu ^2} P. \end{aligned}$$

Hence, *S*(*f*(*t*)) is equivalent to the functional

$$\begin{aligned} \int _{\mathbb {R}^{2n}}\left| \nabla _{x}\left( \frac{f(t)}{f_{\infty }}\right) \right| ^2 f_{\infty }dxdv{+}\int _{\mathbb {R}^{2n}}\nabla ^T_{v}\left( \frac{f(t)}{f_{\infty }}\right) \left( \frac{\partial ^2 V}{\partial x^2}{+} (1{-}\alpha _0) I\right) \nabla _{v}\left( \frac{f(t)}{f_{\infty }}\right) f_{\infty } dxdv. \end{aligned}$$

Subsequently, $\Phi (f(t))$ and the functional on the left hand side of (9) are equivalent. This equivalence and (74) let us obtain (9).

 **Case** (*c*) **and** (*d*),  **estimated decay rate**: Next, we shall estimate $\lambda$ from (73) explicitly, and we shall choose the parameters *a* and $\gamma$ such that $\lambda$ is (rather) large. By (41) and (46), $\eta =\eta (a)$ and $\delta =\delta (a,\gamma )$ are functions of $a\in [c+\frac{\nu ^2}{4}, \infty )\bigcap (\frac{\nu ^2}{4}-\alpha _0, \infty )$ and $\gamma \in [0,\infty ).$ Since $\delta >0,$ and $\eta$ is monotonically increasing up to 2, we have the following uniform estimate and choice of the decay rate:

$$\begin{aligned} 2\lambda {:}{=}\sup _{a\in [c+\frac{\nu ^2}{4}, \infty )\bigcap (\frac{\nu ^2}{4}-\alpha _0, \infty ),\, \gamma \ge 0}\frac{\nu -\tau -\delta (a,\gamma )}{1+\frac{\gamma }{2\eta (a) C_{PI}}}\le \sup _{\gamma \ge 0}\frac{\nu -\tau }{1+\frac{\gamma }{4C_{PI}}}\le \nu -\tau . \end{aligned}$$

Next, we shall estimate this supremum (in fact it is a maximum). First we introduce a new variable $s:=\displaystyle \frac{\gamma \sigma }{4a\sqrt{a+\alpha _0-\frac{\nu ^2}{4}}}\in [0,\infty ),$ then

$$\begin{aligned}\delta (a,\gamma )= \frac{a}{\sqrt{a+\alpha _0-\frac{\nu ^2}{4}}}(\sqrt{1+s^2}-s). \end{aligned}$$

With the notations $\displaystyle A(a):=\frac{1+ a+\alpha _0+ \sqrt{( a+\alpha _0-1)^2+\nu ^2}}{2\sigma C_{PI}}>0$ and $\displaystyle B(a):=\frac{a}{\sqrt{a+\alpha _0-\frac{\nu ^2}{4}}}>0,$ we have

$$\begin{aligned} 2\lambda =\max _{a\in [c+\frac{\nu ^2}{4}, \infty )\bigcap (\frac{\nu ^2}{4}-\alpha _0, \infty ),\, s\ge 0}\frac{\nu -\tau -B(a)(\sqrt{1+s^2}-s)}{1+A(a)B(a)s}. \end{aligned}$$

Next, we shall fix the parameter *a*. To estimate $\lambda$ as accurately as possible, we choose *a* as the argmin of *B*(*a*) such that $\nu -\tau -B(a)(\sqrt{1+s^2}-s)$ is maximal with respect to *a*. The minimal value of *B*(*a*) is

$$\begin{aligned}{} & {} \displaystyle \min _{a\in [c+\frac{\nu ^2}{4}, \infty )\bigcap (\frac{\nu ^2}{4}-\alpha _0, \infty )} B(a) ={\left\{ \begin{array}{ll}\displaystyle B(a_1)=\frac{c+\frac{\nu ^2}{4}}{\sqrt{c+\alpha _0}} \, \, \, \, \, \, \, \, \text { if } \,\, c+2\alpha _0>\frac{\nu ^2}{4}\\ \displaystyle B(a_2)=\sqrt{{\nu ^2}-4\alpha _0} \, \, \text { if } \,\, c+2\alpha _0\le \frac{\nu ^2}{4} \end{array}\right. }, \end{aligned}$$

and this minimum is attained at $a_1{:}{=}{c+\frac{\nu ^2}{4}}$ if $c+2\alpha _0>\frac{\nu ^2}{4}$ (i.e. in Case (*c*)), and $a_2{:}{=}2(\frac{\nu ^2}{4}-\alpha _0)$ if $c+2\alpha _0\le \frac{\nu ^2}{4}$ (i.e. in Case (*d*)).

If $c+2\alpha _0>\frac{\nu ^2}{4},$ then $c>-\alpha _0$ and so *a* varies in

$$\begin{aligned} \Big [c+\frac{\nu ^2}{4}, \infty \Big )\bigcap \Big (\frac{\nu ^2}{4}-\alpha _0, \infty \Big )=\Big [c+\frac{\nu ^2}{4}, \infty \Big )=\Big [a_1, \infty \Big ). \end{aligned}$$

Since *A*(*a*) is increasing, both *A*(*a*) and *B*(*a*) attain their minimal values at $a_1.$ Thus, $a_1$ is optimal, i.e.

$$\begin{aligned} \max _{a}\frac{\nu -\tau -B(a)(\sqrt{1+s^2}-s)}{1+A(a)B(a)s}=\frac{\nu -\tau -B(a_1)(\sqrt{1+s^2}-s)}{1+A(a_1)B(a_1)s}. \end{aligned}$$

If $c+2\alpha _0\le \frac{\nu ^2}{4},$ $a_2=2(\frac{\nu ^2}{4}-\alpha _0)$ may not be optimal as *A*(*a*) does not attain its minimum at this point, i.e.

$$\begin{aligned} \max _{a}\frac{\nu -\tau -B(a)(\sqrt{1+s^2}-s)}{1+A(a)B(a)s}\ge \frac{\nu -\tau -B(a_2)(\sqrt{1+s^2}-s)}{1+A(a_2)B(a_1)s}. \end{aligned}$$

But it is the optimal choice when $s=0$ and so it gives a good approximation if *s* is small. From now on we assume that *a* is fixed as

76 $$\begin{aligned} \displaystyle a{:}{=}{\left\{ \begin{array}{ll} a_1 ={c+\frac{\nu ^2}{4}} \, \, \, \, \, \, \, \, \, \, \, \, \, \text { if } \,\, c+2\alpha _0>\frac{\nu ^2}{4}\\ a_2=2(\frac{\nu ^2}{4}-\alpha _0) \, \, \text { if } \,\, c+2\alpha _0\le \frac{\nu ^2}{4} \end{array}\right. }. \end{aligned}$$

Note that this choice is independent of *s*.

Let $\displaystyle \Lambda (a,s){:}{=}\frac{\nu -\tau -B(a)(\sqrt{1+s^2}-s)}{1+A(a)B(a)s}$ and we seek its maximum with respect to $s\in [0, \infty ).$ We compute

77 $$\begin{aligned}&\partial _s\Lambda (a,s)\nonumber \\&=\frac{B(a)}{(1+A(a)B(a)s)^2\sqrt{s^2+1}}\left( [1-(\nu -\tau -B(a))A(a)]\sqrt{s^2+1}\right. \nonumber \\&\quad \left. -A(a)B(a)(\sqrt{s^2+1}-1)-s \right) . \end{aligned}$$

If $1-(\nu -\tau -B(a))A(a)\le 0,$ then $\partial _s\Lambda (a,s)\le 0$ which implies that $\Lambda (a,s)$ is a decreasing function of *s* and the maximum in $[0,\infty )$ is attained at $s=0.$

If $1-(\nu -\tau -B(a))A(a)>0,$ then $\partial _s\Lambda (a,0)=B(a)[1-(\nu -\tau -B(a))A(a)]>0$ and $\Lambda (a,s)$ is increasing in a neighborhood of $s=0.$ We also see $\partial _s\Lambda (a,s)$ is negative if *s* is large enough (since $\nu -\tau >0$). This means that $\Lambda (a,s)$ starts to grow at $s=0$ and it decreases as $s\rightarrow \infty .$ Therefore, there is a point in $(0,\infty )$ at which $\Lambda (a,s)$ takes its maximum. Setting $\partial _s\Lambda (a,s)=0$ we obtain

$$\begin{aligned}{}[1-(\nu -\tau )A(a)]\sqrt{s^2+1}-s+A(a)B(a)=0. \end{aligned}$$

It has only one solution in $(0,\infty )$ given by

78 $$\begin{aligned} s(a){=}{\left\{ \begin{array}{ll} \frac{A^2(a)B^2(a){-}1}{2A(a)B(a)}&{} \text { if } (\nu {-}\tau )A(a){=}2 \\ \frac{1}{\nu {-}\tau }\left[ \left| \frac{(\nu {-}\tau )A(a){-}1}{(\nu {-}\tau )A(a){-}2}\right| \sqrt{B^2(a) {+}2(\nu {-}\tau )A^{-1}(a)-(\nu -\tau )^2}-\frac{B(a)}{(\nu -\tau )A(a)-2}\right] &{} \text { if } (\nu -\tau )A(a)\ne 2 \end{array}\right. }\nonumber \\ \end{aligned}$$

and at this point $\Lambda (a,s)$ attains its maximum with respect to *s*.

Considering the computations above, we conclude that the decay rate can be estimated by:

79 $$\begin{aligned} \displaystyle 2\lambda ={\left\{ \begin{array}{ll} \nu -\tau -B(a)&{} \text { if } \nu -\tau \ge A^{-1}(a)+B(a)\\ \frac{\nu -\tau -B(a)(\sqrt{1+s^2(a)}-s(a))}{1+A(a)B(a)s(a)} &{} \text { if } \nu -\tau < A^{-1}(a)+B(a) \end{array}\right. }, \end{aligned}$$

where two cases correspond to the two cases discussed after (77). Moreover, *a* and *s*(*a*) are defined in (76) and (78), respectively. If we denote $A_1:=A(a_1),$ $A_2:=A(a_2),$ $s_1:=s(a_1)$ and $s_2:=s(a_2)$ and take into account that $B(a_1)=\frac{c+\frac{\nu ^2}{4}}{\sqrt{c+\alpha _0}}$ and $B(a_2)=\sqrt{{\nu ^2}-4\alpha _0},$ we obtain the explicit decay rates stated in the theorem.

 **Case** (*e*) :  Let *V*(*x*) be a quadratic function of *x* and $\frac{\partial ^2 V}{\partial x^2}$ be positive definite. Then, $\frac{\partial ^2 (\partial _{x_i}V)}{\partial x^2}$ are zero matrices for all $i\in \{1,...,n\}.$ Thus, *V* satisfies Assumption 2.2 with $\tau =0,$ $-c=\alpha _0>0.$

If $\alpha _0<\frac{\nu ^2}{4},$ then $c+2\alpha _0=\alpha _0<\frac{\nu ^2}{4}$ which falls into Case (*d*).

The constant in the Poincaré inequality (5) equals $C_{PI}=\frac{\nu }{\sigma }\min \{1, \alpha _0\}$ (see [4]). It lets us compute $A_2^{-1}$ explicitly:

$$\begin{aligned} \displaystyle A_2^{-1}=\frac{2\nu \min \{1, \alpha _0\}}{1+ \frac{\nu ^2}{2}-\alpha _0+ \sqrt{\big ( \frac{\nu ^2}{2}-\alpha _0-1\big )^2+\nu ^2}}. \end{aligned}$$

In Appendix 6.3 we prove the following inequality:

80 $$\begin{aligned} \nu \ge A^{-1}_2+\sqrt{{\nu ^2}-4\alpha _0}. \end{aligned}$$

Thus Case (*d*) implies

81 $$\begin{aligned} \lambda = \frac{\nu -\sqrt{{\nu ^2}-4\alpha _0}}{2}. \end{aligned}$$

If $\alpha _0\ge \frac{\nu ^2}{4},$ the decay rate is explicit by Case (*a*) and Case (*b*) : 

82 $$\begin{aligned} \lambda ={\left\{ \begin{array}{ll} {\frac{\nu }{2}}&{} \text { if } \alpha _0>\frac{\nu ^2}{4}\\ \frac{ \nu -\varepsilon }{2}&{} \text { if } \alpha _0=\frac{\nu ^2}{4}, \text { for any } \varepsilon \in (0,\nu ) \end{array}\right. }. \end{aligned}$$

We now prove that the decay rates in (81) and (82) are sharp: From Corollary 2.8

$$\begin{aligned}{} & {} \int _{\mathbb {R}^{2n}}\left( \frac{f(t)}{f_{\infty }}-1\right) ^2 f_{\infty }dxdv \\{} & {} \quad \le C e^{-2\lambda t} \int _{\mathbb {R}^{2n}} \left( \frac{f_0}{f_{\infty }} -1\right) ^2 \left( \left| \left| \frac{\partial ^2V}{\partial x^2}\right| \right| ^2+1\right) f_{\infty }dxdv,\, \, \, \, \, \, \forall t\ge t_0 \end{aligned}$$

holds with the same $\lambda$ given in (81) and (82). Since $\left| \left| \frac{\partial ^2V}{\partial x^2}\right| \right| +1$ is constant, this estimate implies

83 $$\begin{aligned} \sup _{1\ne \frac{f_0}{f_{\infty }} \in L^2(\mathbb {R}^d, f_{\infty })}\frac{||f(t)/f_{\infty }-1||_{L^2(\mathbb {R}^d, f_{\infty })}}{||f_0/f_{\infty }-1||_{L^2(\mathbb {R}^d, f_{\infty })}}\le {\tilde{C}} e^{-\lambda t},\, \, \, \, \, \, \forall t\ge t_0 \end{aligned}$$

for some constant ${\tilde{C}}>0.$ On the one hand this means that the estimated decay rate $\lambda$ can not be larger than the (true) decay rate of the propagator norm given on the left hand side of (83). On the other hand, Proposition 2.5 gives the sharp decay rates for this propagator norm. The decay rates in (81) and (82) coincide with the ones in Proposition 2.5 except in the case of $\alpha _0= \frac{\nu ^2}{4}.$ Thus, the exponential decay rates in Case (*a*) and Case (*d*) are sharp. When $\alpha _0= \frac{\nu ^2}{4},$ Proposition 2.5 provides the sharp decay $(1+t)e^{-\frac{\nu }{2} t}$ for the propagator norm. Hence, (9) can hold with rates $\lambda =\frac{\nu -\varepsilon }{2}$ for any small fixed $\varepsilon \in (0,\nu ),$ but it does not hold for $\varepsilon =0.$ $\square$

### Proof of Proposition 2.5

#### Proof of Proposition 2.5

Let *V* be a quadratic polynomial and $\frac{\partial ^2 V}{\partial x^2}{=}{:}M^{-1} \in \mathbb {R}^{n\times n}$ be positive definite. Then there are $x_0\in \mathbb {R}^n$ and $\mathcal {C}\in \mathbb {R}$ such that $V(x)=\frac{{(x-x_0)}^T M^{-1}(x-x_0)}{2}+\mathcal {C},$ $\forall x \in \mathbb {R}^n.$ Since the change $x \rightarrow x+x_0$ does not affect the supremum in (10) and only the gradient of *V* appears in (1), without loss of generality we assume that $x_0=0$ and $\mathcal {C}=0.$

 **Step 1, reformulation as an ODE-problem**: To this end we use Theorem 3.2. We check the conditions of this theorem for the kinetic Fokker–Planck equation. With the notation $$\xi =\begin{pmatrix} x\\ v \end{pmatrix},$$ we write

84 $$\begin{aligned} E(\xi ){=}\frac{\nu }{\sigma }\left( V(x){+}\frac{|v|^2}{2}\right) {=}\frac{\nu }{\sigma }\left( \frac{{x}^T M^{{-}1} x}{2}{+}\frac{|v|^2}{2}\right) {=}\frac{1}{2}\xi ^T\begin{pmatrix} \frac{\nu }{\sigma }M^{-1}&{} 0\\ 0&{}\frac{\nu }{\sigma }I \end{pmatrix} \xi =\frac{\xi ^TK^{-1}\xi }{2}\nonumber \\ \end{aligned}$$

with $$K^{-1}:=\frac{\nu }{\sigma }\begin{pmatrix} M^{-1}&{} 0\\ 0&{}I \end{pmatrix}.$$

From (25) we see that $\text {Ker}D=\{(\psi ,0)^T: \, \, \psi \in \mathbb {R}^n\}.$ Let $(\psi ,0)^T\in \text {Ker}D,$ then its image under $K^{-1}(D-R)$ is

$$\begin{aligned} K^{-1}(D-R)\begin{pmatrix} \psi \\ 0 \end{pmatrix}=\begin{pmatrix} 0 &{} M^{-1}\\ -I &{} \nu I \end{pmatrix} \begin{pmatrix} \psi \\ 0 \end{pmatrix}=\begin{pmatrix} 0\\ psi \end{pmatrix} \end{aligned}$$

and it is in $\text {Ker}D$ iff $\psi =0.$ Therefore, there is no non-trivial $K^{-1}(D-R)$-invariant subspace of $\text {Ker}D.$ Next we compute the eigenvalues $\beta$ of $$K^{-1/2}(D+R)K^{-1/2}=\begin{pmatrix} 0&{}-M^{-1/2}\\ M^{-1/2}&{}\nu I \end{pmatrix}:$$

$$\begin{aligned} \begin{vmatrix} -\beta I&-M^{-1/2}\\ M^{-1/2}&(\nu -\beta )I \end{vmatrix}=\begin{vmatrix} -\beta I&0\\ M^{-1/2}&(\nu -\beta )I-\beta ^{-1}M^{-1} \end{vmatrix}\\= \text {det}(\beta (\beta -\nu )I+M^{-1})=\prod _{i=1}^n(\beta ^2-\nu \beta +\alpha _i)=0, \end{aligned}$$

where $\alpha _i,$ $i\in \{1,...,n\}$ denote the eigenvalues of $M^{-1}.$ By solving the latter equation, we find that the eigenvalues of $K^{-1/2}(D+R)K^{-1/2}$ are $\beta ^{-}_{i}=\frac{\nu -\sqrt{\nu ^2-4\alpha _i}}{2},$ $\beta ^{+}_{i}=\frac{\nu +\sqrt{\nu ^2-4\alpha _i}}{2},$ $i\in \{1,...,n\}.$ If $\alpha _0>0$ is the smallest eigenvalue of $M^{-1},$ then

$$\begin{aligned} \mu {:}{=}\min _i\{\textsf {Re}(\beta _i): \beta _i \text { is an eigenvalue of } K^{-1/2}(D+R)K^{-1/2}\}={\left\{ \begin{array}{ll} \frac{\nu }{2}&{} \text { if } \alpha _0\ge \frac{\nu ^2}{4}\\ \frac{\nu -\sqrt{\nu ^2-4\alpha _0}}{2} &{} \text { if } \alpha _0<\frac{\nu ^2}{4} \end{array}\right. }. \end{aligned}$$

Hence $\mu$ is positive, so $K^{-1/2}(D+R)K^{-1/2}$ and $(D+R)K^{-1}$ are positive stable. Therefore, Theorem 3.2 applies to the kinetic Fokker–Planck equation.

 **Step 2, decay rates of the ODE-solution**: We consider the ODE

$$\begin{aligned} {\dot{\xi }}(t)=-K^{-1/2}(D+R)K^{-1/2}\xi \end{aligned}$$

with the initial data $\xi (0)=\xi _0.$ Since $K^{-1/2}(D+R)K^{-1/2}$ is positive stable, the solution ${\xi }(t)$ is stable. To quantify the decay rate, we continue to analyze the eigenvalues of $K^{-1/2}(D+R)K^{-1/2}.$ Let $m_i$ be the multiplicity of $\alpha _i>0$ as an eigenvalue of $M^{-1}$ (now the $\alpha _i$ with $i\in \{1,...,{\tilde{n}}\}$ are labeled without multiplicity). Since $M^{-1}$ is symmetric, there are linearly independent eigenvectors $\psi _{ij}\in \mathbb {R}^n, \, \, j \in \{1,..., m_i\}$ of $M^{-1}$ corresponding to $\alpha _i.$ Then we can check that the vectors

85 $$\begin{aligned} \begin{pmatrix} -\frac{\alpha _i^{1/2}}{\beta ^{-}_{i}}\psi _{ij}\\ \psi _{ij} \end{pmatrix}\in \mathbb {R}^{2n}, \, \, j \in \{1,..., m_i\} \end{aligned}$$

are linearly independent eigenvectors of $K^{-1/2}(D+R)K^{-1/2}$ corresponding to $\beta ^{-}_{i},$ $i\in \{1,...,{\tilde{n}}\}.$ Moreover, these vectors form a basis of the space of eigenvectors corresponding to $\beta ^{-}_{i}.$ Similarly, the vectors

86 $$\begin{aligned} \begin{pmatrix} -\frac{\alpha _i^{1/2}}{\beta ^{+}_{i}}\psi _{ij}\\ \psi _{ij} \end{pmatrix}\in \mathbb {R}^{2n}, \, \, j \in \{1,..., m_i\}. \end{aligned}$$

satisfy the same property for $\beta ^{+}_{i}.$

If $\alpha _i\ne \frac{\nu ^2}{4}$ for all $i\in \{1,...,{\tilde{n}}\}$ (i.e., $\beta ^{-}_{i}\ne \beta ^{+}_{i}),$ the algebraic multiplicities of $\beta ^{-}_i$ and $\beta ^{+}_i$ are equal to $m_i.$ Then $\beta ^{-}_i$ (resp. $\beta ^{+}_i$) has $m_i$ eigenvectors given by (85) (resp. (86)). Thus, the geometric multiplicities of $\beta ^{-}_i$ and $\beta ^{+}_i$ also equal $m_i.$ In particular, $K^{-1/2}(D+R)K^{-1/2}$ is diagonalizable.

If $\alpha _{i_0}=\frac{\nu ^2}{4}$ for some $i_0\in \{1,...,{\tilde{n}}\},$ then the algebraic multiplicity of $\beta ^{-}_{i_0}=\beta ^{+}_{i_0}=\frac{\nu }{2}$ equals $2m_{i_0}.$ Since the vectors (85) and (86) coincide in this case, the geometric multiplicity of $\frac{\nu }{2}$ equals $m_{i_0}.$ Thus, in this case, $\frac{\nu }{2}$ is a defective^3^ eigenvalue of $K^{-1/2}(D+R)K^{-1/2}$ with the corresponding eigenvectors

87 $$\begin{aligned} \begin{pmatrix} -\psi _{i_0 j}\\ \psi _{i_0 j} \end{pmatrix}\in \mathbb {R}^{2n}, \, \, j \in \{1,..., m_{i_0}\}. \end{aligned}$$

By solving the following linear system (with respect to $\xi$)

$$\begin{aligned} K^{-1/2}(D+R)K^{-1/2}\xi -\frac{\nu }{2}\xi =\begin{pmatrix} -\frac{\nu }{2} I&{}-M^{-1/2}\\ M^{-1/2}&{}\frac{\nu }{2}I \end{pmatrix}\xi =\begin{pmatrix} -\psi _{i_0 j}\\ \psi _{i_0 j} \end{pmatrix}, \, \, \, \, \xi \in \mathbb {R}^{2d}, \end{aligned}$$

we find that the solution $$ \xi = \begin{pmatrix} 0\\ \frac{2}{\nu }\psi _{i_0 j} \end{pmatrix} $$ is a generalized eigenvector of $\frac{\nu }{2}$ corresponding to the eigenvector $$\begin{pmatrix} -\psi _{i_0 j}\\ \psi _{i_0 j} \end{pmatrix}.$$ Since $\psi _{i_0 j},$ $j \in \{1,..., m_{i_0}\}$ are linearly independent, the vectors

88 $$\begin{aligned} \begin{pmatrix} 0\\ \frac{2}{\nu }\psi _{i_0 j} \end{pmatrix}, \, \, j \in \{1,..., m_{i_0}\} \end{aligned}$$

form a set of linearly independent generalized eigenvectors of $\frac{\nu }{2}.$ Since the vectors in (87) and (88) are linearly independent and their total number equals $2m_{i_0}$ (which is the algebraic multiplicity of $\frac{\nu }{2}$), we conclude that each eigenvector of $\frac{\nu }{2}$ has only one generalized eigenvector. Therefore, all Jordan blocks associated to $\frac{\nu }{2}$ have the same size $2\times 2.$ In particular, if $\alpha _0=\frac{\nu ^2}{4},$ then the eigenvalue $\mu =\frac{\nu }{2}$ is defective and the maximal size of the Jordan blocks associated to $\frac{\nu }{2}$ is 2.

Then, the classical stability theory for ODEs shows that

$$\begin{aligned}{} & {} \sup _{1\ne \frac{f_0}{f_{\infty }} \in L^2(\mathbb {R}^d, f_{\infty })}\frac{||f(t)/f_{\infty }-1||_{L^2(\mathbb {R}^d, f_{\infty })}}{||f_0/f_{\infty }-1||_{L^2(\mathbb {R}^d, f_{\infty })}}\\{} & {} \quad =\sup _{0\ne \xi _0\in \mathbb {R}^d}\frac{||\xi (t)||_2}{||\xi _0||_2}\asymp {\left\{ \begin{array}{ll} e^{-\frac{\nu }{2}t},&{} \text { if } \alpha _0>\frac{\nu ^2}{4} \\ (1+ t)e^{-\frac{\nu }{2}t},&{} \text { if } \alpha _0=\frac{\nu ^2}{4}\\ e^{-{\frac{\nu -\sqrt{\nu ^2-4\alpha _0}}{2}t}},&{} \text { if } \alpha _0<\frac{\nu ^2}{4} \end{array}\right. } \end{aligned}$$

as $$ t\rightarrow \infty .$$ $$\square $$

#### Remark 5.1

With the eigenvalues of $C{:}{=}(D+R)K^{-1}$ (see (24), (84)) obtained at the end of Step 1 in the above proof, the sharpness of the decay rate $\mu$ in the cases 1 and 3 of (10) would also follow from [3, Theorem 6.1].

### Proof of Theorem 2.7 and Corollary 2.8

#### Proof of Theorem 2.7

**Step 1, an auxiliary inequality**: As we assume the matrix (6) is positive semi-definite, then the following submatrices of (6) are positive semi-definite:

$$\begin{aligned} Y_k{:}{=}\begin{pmatrix} \nu \left( \frac{\partial ^2 V}{\partial x^2}+cI\right) &{}-\frac{1}{2}\frac{\partial ^2(\partial _{x_k}V)}{\partial x^2} \\ -\frac{1}{2}\frac{\partial ^2(\partial _{x_k}V)}{\partial x^2}&{}\frac{\tau \nu }{2\sigma }\left( \frac{\partial ^2 V}{\partial x^2}+cI\right) \end{pmatrix} \in \mathbb {R}^{2n\times 2n}, \, \, \, \, \, \, k\in \{1,...,n\}. \end{aligned}$$

Letting $\delta >0,$ we consider

$$\begin{aligned} X_{\delta }{:}{=}\begin{pmatrix}I&{}\delta I\\ \delta I&{}\delta ^2 I \end{pmatrix} \otimes \left( \frac{\partial ^2 V}{\partial x^2}+cI\right) =\begin{pmatrix} \frac{\partial ^2 V}{\partial x^2}+cI&{}\delta \frac{\partial ^2 V}{\partial x^2}+\delta cI \\ \delta \frac{\partial ^2 V}{\partial x^2}+\delta cI&{}\delta ^2 \frac{\partial ^2 V}{\partial x^2}+\delta ^2 cI \end{pmatrix}\in \mathbb {R}^{2n\times 2n}. \end{aligned}$$

$X_{\delta }$ is positive semi-definite as it is the Kronecker product [27, Corollary 4.2.13] of two positive semi-definite matrices. Hence, we have for all $k \in \{1,...,n\}:$

$$\begin{aligned} \textrm{Tr}(X^{1/2}_{\delta }Y_kX^{1/2}_{\delta }){} & {} =\textrm{Tr}(X_{\delta }Y_k)\\{} & {} =(\nu +\delta ^2\frac{ \tau \nu }{2\sigma }) \textrm{Tr} \left[ \left( \frac{\partial ^2 V}{\partial x^2}+c I\right) ^2\right] \\{} & {} \quad -\delta \textrm{Tr}\left[ \left( \frac{\partial ^2V}{\partial x^2}+cI\right) \frac{\partial ^2(\partial _{x_k}V)}{\partial x^2}\right] \ge 0. \end{aligned}$$

This implies

89 $$\begin{aligned} \frac{2\sigma \nu +\delta ^2 \tau \nu }{2\sigma \delta } \textrm{Tr} \left[ \left( \frac{\partial ^2 V}{\partial x^2}+c I\right) ^2\right] \ge \textrm{Tr}\left[ \left( \frac{\partial ^2V}{\partial x^2}+c I\right) \frac{\partial ^2(\partial _{x_k}V)}{\partial x^2}\right] \end{aligned}$$

and by minimizing the constant on the left hand side of (89) with respect to $\delta$ (i.e., by choosing $\delta =\sqrt{\frac{2\sigma }{\tau }}$), we obtain

90 $$\begin{aligned} \sqrt{\frac{2\tau \nu ^2}{\sigma }}\textrm{Tr} \left[ \left( \frac{\partial ^2 V(x)}{\partial x^2}{+}c I\right) ^2\right] {\ge } \textrm{Tr}\left[ \left( \frac{\partial ^2V(x)}{\partial x^2}{+}cI\right) \frac{\partial ^2(\partial _{x_k}V(x))}{\partial x^2}\right] \, \, \, \, \, \, \, \text {for all} \,\, \, \, x \in \mathbb {R}^n.\nonumber \\ \end{aligned}$$

$$\square $$

 **Step 2, growth estimate for the r.h.s. of** (11), (12): We denote $\displaystyle u_1{:}{=}\nabla _x \left( \frac{f(t)}{f_{\infty }}\right) ,$ $\displaystyle u_2{:}{=}\nabla _v \left( \frac{f(t)}{f_{\infty }}\right) ,$ and $$u{:}{=}\begin{pmatrix} u_1\\ u_2 \end{pmatrix}.$$ Since $\displaystyle \frac{f(t)}{f_{\infty }}-1$ satisfies

$$\begin{aligned} \partial _t \left( \frac{f(t)}{f_{\infty }}-1\right){} & {} =-v \cdot \nabla _x \left( \frac{f(t)}{f_{\infty }}-1\right) +\nabla _x V \cdot \nabla _v \left( \frac{f(t)}{f_{\infty }}-1\right) \\{} & {} \quad +\sigma \Delta _v \left( \frac{f(t)}{f_{\infty }}-1\right) -\nu v\cdot \nabla _v \left( \frac{f(t)}{f_{\infty }}-1\right) \end{aligned}$$

and by integrating by parts, we obtain

91 $$\begin{aligned} \frac{d}{dt} \displaystyle \int _{\mathbb {R}^{2n}} \left( \frac{f(t)}{f_{\infty }} -1\right) ^2f_{\infty }dxdv=-2\sigma \displaystyle \int _{\mathbb {R}^{2n}} |u_2|^2 f_{\infty }dxdv. \end{aligned}$$

Next, we compute (with $||\cdot ||$ denoting the Frobenius norm)

92 $$\begin{aligned}&\frac{d}{dt} \displaystyle \int _{\mathbb {R}^{2n}} \left( \frac{f(t)}{f_{\infty }} -1\right) ^2 \left| \left| \frac{\partial ^2V}{\partial x^2}+c I\right| \right| ^2 f_{\infty }dxdv \nonumber \\&\quad =2\int _{\mathbb {R}^{2n}} \left( \frac{f(t)}{f_{\infty }}-1 \right) \partial _t \left( \frac{f(t)}{f_{\infty }}-1\right) \left| \left| \frac{\partial ^2V}{\partial x^2}+c I\right| \right| ^2 f_{\infty }dxdv \nonumber \\&\quad =2\int _{\mathbb {R}^{2n}} \left( \frac{f(t)}{f_{\infty }} -1\right) \left[ -v \cdot \nabla _x \left( \frac{f(t)}{f_{\infty }}-1\right) \right. \nonumber \\&\qquad \left. +\nabla _x V \cdot \nabla _v \left( \frac{f(t)}{f_{\infty }}-1\right) \right] \left| \left| \frac{\partial ^2V}{\partial x^2}+c I\right| \right| ^2 f_{\infty }dxdv \nonumber \\&\qquad {+}2\int _{\mathbb {R}^{2n}} \left( \frac{f(t)}{f_{\infty }} {-}1\right) \left[ \sigma \Delta _v \left( \frac{f(t)}{f_{\infty }}{-}1\right) {-}\nu v\cdot \nabla _v \left( \frac{f(t)}{f_{\infty }}{-}1\right) \right] \left| \left| \frac{\partial ^2V}{\partial x^2}{+}c I\right| \right| ^2 f_{\infty }dxdv. \end{aligned}$$

Integrating by parts with respect to *v*,  we obtain

93 $$\begin{aligned}&2\int _{\mathbb {R}^{2n}} \left( \frac{f(t)}{f_{\infty }} -1\right) \left[ \sigma \Delta _v \left( \frac{f(t)}{f_{\infty }}-1\right) -\nu v\cdot \nabla _v \left( \frac{f(t)}{f_{\infty }}-1\right) \right] \left| \left| \frac{\partial ^2V}{\partial x^2}+c I\right| \right| ^2 f_{\infty }dxdv\nonumber \\&\quad = -2\sigma \displaystyle \int _{\mathbb {R}^{2n}} |u_2|^2 \left| \left| \frac{\partial ^2V}{\partial x^2}+c I\right| \right| ^2 f_{\infty }dxdv. \end{aligned}$$

Next, we work on the term in the second line of (92):

94 $$\begin{aligned}&2\int _{\mathbb {R}^{2n}} \left( \frac{f(t)}{f_{\infty }} {-}1\right) \left[ {-}v \cdot \nabla _x \left( \frac{f(t)}{f_{\infty }}{-}1\right) {+}\nabla _x V \cdot \nabla _v \left( \frac{f(t)}{f_{\infty }}{-}1\right) \right] \left| \left| \frac{\partial ^2V}{\partial x^2}{+}c I\right| \right| ^2 f_{\infty }dxdv\nonumber \\&\quad =\int _{\mathbb {R}^{2n}} \left( -v \cdot \nabla _x \left( \frac{f(t)}{f_{\infty }}-1\right) ^2+\nabla _x V \cdot \nabla _v \left( \frac{f(t)}{f_{\infty }}-1\right) ^2\right) \left| \left| \frac{\partial ^2V}{\partial x^2}+c I\right| \right| ^2 f_{\infty }dxdv \nonumber \\&\quad =\int _{\mathbb {R}^{2n}} \left( \frac{f(t)}{f_{\infty }}{-}1\right) ^2 \left[ v \cdot \nabla _x \left( \left| \left| \frac{\partial ^2V}{\partial x^2}{+}c I\right| \right| ^2 f_{\infty }\right) {-}\nabla _x V \cdot \nabla _v \left( \left| \left| \frac{\partial ^2V}{\partial x^2}{+}c I\right| \right| ^2 f_{\infty }\right) \right] dxdv \nonumber \\&\quad =\int _{\mathbb {R}^{2n}} \left( \frac{f(t)}{f_{\infty }}-1\right) ^2 v \cdot \nabla _x \left( \left| \left| \frac{\partial ^2V}{\partial x^2}+c I\right| \right| ^2\right) f_{\infty }dxdv \nonumber \\&\quad = \frac{2\sigma }{\nu }\int _{\mathbb {R}^{2n}} \left( \frac{f(t)}{f_{\infty }} -1\right) u_2\cdot \nabla _x\left( \left| \left| \frac{\partial ^2V}{\partial x^2}+c I\right| \right| ^2\right) f_{\infty }dxdv \nonumber \\&\quad =\frac{2\sigma }{\nu }\int _{\mathbb {R}^{2n}} \left( \frac{f(t)}{f_{\infty }} -1\right) \sum _{k=1}^n u_{2,k} \partial _{x_k}\left( \left| \left| \frac{\partial ^2V}{\partial x^2}+c I\right| \right| ^2\right) f_{\infty }dxdv \nonumber \\&\quad =\frac{4\sigma }{\nu }\int _{\mathbb {R}^{2n}} \left( \frac{f(t)}{f_{\infty }} -1\right) \left\{ \sum _{k=1}^n u_{2,k}\sum _{i,j=1}^n(\partial ^2_{x_i x_j}V+\delta _{ij}c)\partial ^2_{x_i x_j}(\partial _{x_k}V)\right\} f_{\infty }dxdv, \end{aligned}$$

where we integrated by parts twice, and used $-\frac{\nu }{\sigma }vf_{\infty }=\nabla _vf_{\infty }$ and the notations

$$\begin{aligned} u_{2,k}{:}{=}\partial _{v_k} \left( \frac{f(t)}{f_{\infty }} \right) \,\,\, \, \text { and } \, \,\, \, \delta _{ij}{:}{=}{\left\{ \begin{array}{ll} 1 \, \, \, \, \text { if }\,\, i=j\\ 0 \, \, \, \, \text { if }\,\, i\ne j\end{array}\right. }. \end{aligned}$$

Using the identity

$$\begin{aligned} \displaystyle \sum _{i,j=1}^n(\partial ^2_{x_i x_j}V+\delta _{ij}c)\partial ^2_{x_i x_j}(\partial _{x_k}V)=\textrm{Tr}\left[ \left( \frac{\partial ^2V}{\partial x^2}+cI\right) \frac{\partial ^2(\partial _{x_k}V)}{\partial x^2}\right] , \end{aligned}$$

the estimate (90), and the discrete Hölder inequality, (94) can be estimated as

95 $$\begin{aligned}&\frac{4\sigma }{\nu }\int _{\mathbb {R}^{2n}} \left( \frac{f(t)}{f_{\infty }} -1\right) \left\{ \sum _{i,j,k=1}^n u_{2,k}(\partial ^2_{x_i x_j}V+\delta _{ij}c)\partial ^2_{x_i x_j}(\partial _{x_k}V)\right\} f_{\infty }dxdv \nonumber \\&\quad =\frac{4\sigma }{\nu }\int _{\mathbb {R}^{2n}} \left( \frac{f(t)}{f_{\infty }} -1\right) \left\{ \sum _{k=1}^n u_{2,k}\textrm{Tr}\left[ \left( \frac{\partial ^2V}{\partial x^2}+c I\right) \frac{\partial ^2(\partial _{x_k}V)}{\partial x^2}\right] \right\} f_{\infty }dxdv\nonumber \\&\quad \le 4\sqrt{2 \sigma \tau }\int _{\mathbb {R}^{2n}} \left( \frac{f(t)}{f_{\infty }} -1\right) \left\{ \sum _{k=1}^n |u_{2,k}|\textrm{Tr}\left[ \left( \frac{\partial ^2V}{\partial x^2}+c I\right) ^2\right] \right\} f_{\infty }dxdv \nonumber \\&\quad \le 4\sqrt{2 \sigma \tau n}\int _{\mathbb {R}^{2n}} \left( \frac{f(t)}{f_{\infty }} -1\right) |u_{2}|\textrm{Tr}\left[ \left( \frac{\partial ^2V}{\partial x^2}+c I\right) ^2\right] f_{\infty }dxdv \nonumber \\&\quad \le \sigma \int _{\mathbb {R}^{2n}} |u_{2}|^2\textrm{Tr}\left[ \left( \frac{\partial ^2V}{\partial x^2}+c I\right) ^2\right] f_{\infty }dxdv\nonumber \\&\qquad +8\tau n\int _{\mathbb {R}^{2n}} \left( \frac{f(t)}{f_{\infty }}-1 \right) ^2 \textrm{Tr}\left[ \left( \frac{\partial ^2V}{\partial x^2}+c I\right) ^2\right] f_{\infty }dxdv. \end{aligned}$$

Combining the equations from (92) to (95) and the identity

$$\begin{aligned} \displaystyle \left| \left| \frac{\partial ^2V}{\partial x^2}+c I\right| \right| ^2=\textrm{Tr}\left[ \left( \frac{\partial ^2V}{\partial x^2}+c I\right) ^2\right] , \end{aligned}$$

we get

96 $$\begin{aligned}&\frac{d}{dt} \displaystyle \int _{\mathbb {R}^{2n}} \left( \frac{f(t)}{f_{\infty }} -1\right) ^2 \left| \left| \frac{\partial ^2V}{\partial x^2}+c I\right| \right| ^2 f_{\infty }dxdv \nonumber \\&\quad \le -\sigma \int _{\mathbb {R}^{2n}} |u_{2}|^2\left| \left| \frac{\partial ^2V}{\partial x^2}{+}cI\right| \right| ^2 f_{\infty }dxdv{+}8\tau n\int _{\mathbb {R}^{2n}} \left( \frac{f(t)}{f_{\infty }} {-}1\right) ^2 \left| \left| \frac{\partial ^2V}{\partial x^2}{+}c I\right| \right| ^2 f_{\infty }dxdv. \end{aligned}$$

(96) can be reformulated as

97 $$\begin{aligned}&\frac{d}{dt}\left( e^{-8\tau n t}\displaystyle \int _{\mathbb {R}^{2n}} \left( \frac{f(t)}{f_{\infty }} -1\right) ^2 \left| \left| \frac{\partial ^2V}{\partial x^2}+c I\right| \right| ^2 f_{\infty }dxdv \right) \nonumber \\&\quad \le -\sigma e^{-8\tau n t} \int _{\mathbb {R}^{2n}} |u_{2}|^2\left| \left| \frac{\partial ^2V}{\partial x^2}+c I\right| \right| ^2 f_{\infty }dxdv. \end{aligned}$$

 **Step 3,** *t*-**dependent functional** $\Psi$: In order to prove the short-time regularization of (11) and (12) we introduce now an auxiliary functional that depends explicitly on time. Our strategy is the generalization of the approach in [31, Theorem A.12], [23, Theorem 1.1], [3, Theorem 4.8].

For $t \in (0,t_0],$ we consider the following functional

98 $$\begin{aligned} \displaystyle \Psi (t,f(t)){} & {} {:}{=}\displaystyle \int _{\mathbb {R}^{2n}} \left( \frac{f(t)}{f_{\infty }} -1\right) ^2 \left( \gamma _1 e^{-8\tau n t}\left| \left| \frac{\partial ^2V}{\partial x^2}+c I\right| \right| ^2+\gamma _2\right) f_{\infty }dxdv\nonumber \\{} & {} \quad +\int _{\mathbb {R}^{2n}} u^TP u f_{\infty }dxdv, \end{aligned}$$

with the *t*- and *x*-dependent matrix in $\mathbb {R}^{2n\times 2n},$

99 $$\begin{aligned} P=P(t,x){:}{=}\begin{pmatrix} 2\varepsilon ^3 t^3I&{}\varepsilon ^2 t^2I\\ \varepsilon ^2 t^2I&{} 2\varepsilon tI+t(\frac{\partial ^2V}{\partial x^2}+cI) \end{pmatrix}. \end{aligned}$$

$\varepsilon ,$ $\gamma _1,$ and $\gamma _2$ are positive constants which we shall fix later. We note that, for all $t\in (0,t_0],$

100 $$\begin{aligned} P(t,x)\ge \begin{pmatrix} \varepsilon ^3 t^3I&{}0\\ 0&{} t(\frac{\partial ^2V}{\partial x^2}+cI)+\varepsilon tI \end{pmatrix}>\begin{pmatrix} \varepsilon ^3 t^3I&{}0\\ 0&{} t(\frac{\partial ^2V}{\partial x^2}+cI) \end{pmatrix}\ge 0 \end{aligned}$$

as $\frac{\partial ^2V}{\partial x^2}+cI$ is positive semi-definite. Thus, $\Psi (t,f(t))$ is non-negative and satisfies

101 $$\begin{aligned} \displaystyle \Psi (t, f(t))&\ge \displaystyle \displaystyle \int _{\mathbb {R}^{2n}} \left( \frac{f(t)}{f_{\infty }} -1\right) ^2 \left( \gamma _1 e^{-8\tau n t} \left| \left| \frac{\partial ^2V}{\partial x^2}+c I\right| \right| ^2+\gamma _2\right) f_{\infty }dxdv\nonumber \\&\quad +\varepsilon ^3t^3\displaystyle \int _{\mathbb {R}^{2n}}|u_1|^2 f_{\infty } dx dv\nonumber \\&\quad + t\int _{\mathbb {R}^{2n}} u_2^T \left( \frac{\partial ^2V}{\partial x^2}+(c+\varepsilon )I\right) u_2 f_{\infty } dxdv. \end{aligned}$$

Our goal is to show that $\Psi (t, f(t))$ decreases. To this end we estimate the time derivative of the second term in (98). First, (27) yields

102 $$\begin{aligned}&\frac{d}{dt}\int _{\mathbb {R}^{2n}}u^TPuf_{\infty }dxdv \nonumber \\&\quad =-2\sigma \int _{\mathbb {R}^{2n}}\left\{ \sum _{i=1}^n (\partial _{v_i}u)^TP\partial _{v_i}u\right\} f_{\infty }dxdv-\int _{\mathbb {R}^{2n}} u^T\left\{ QP+PQ^T-\partial _t P\right\} u f_{\infty }dxdv\nonumber \\&\qquad -\int _{\mathbb {R}^{2n}} u^T\left\{ [\nabla _x V\cdot \nabla _v-v\cdot \nabla _x+\nu v\cdot \nabla _v-\sigma \Delta _v]P\right\} u f_{\infty }dxdv, \end{aligned}$$

with $$Q= \begin{pmatrix} 0&{}I\\ -\frac{\partial ^2 V(x)}{\partial x^2}&{}\nu I \end{pmatrix}.$$ We consider each terms of (102). Because of (100), the first term can be estimated as

103 $$\begin{aligned}{} & {} -2\sigma \int _{\mathbb {R}^{2n}}\left\{ \sum _{i=1}^n (\partial _{v_i}u)^TP\partial _{v_i}u\right\} f_{\infty }dxdv\nonumber \\{} & {} \quad \le -2t\sigma \int _{\mathbb {R}^{2n}}\left\{ \sum _{i=1}^n (\partial _{v_i}u_2)^T\left( \frac{\partial ^2V}{\partial x^2}+c I\right) \partial _{v_i}u_2\right\} f_{\infty }dxdv. \end{aligned}$$

For the third term of (102) we have

$$\begin{aligned}{}[\nabla _x V\cdot \nabla _v-v\cdot \nabla _x+\nu v\cdot \nabla _v-\sigma \Delta _v]P=\begin{pmatrix} 0&{}0\\ 0&{} -t\frac{\partial ^2 (v\cdot \nabla _x V)}{\partial x^2} \end{pmatrix} \end{aligned}$$

and using $vf_{\infty }=-\frac{\sigma }{\nu }\nabla _v f_{\infty }$ yields

104 $$\begin{aligned}&-\int _{\mathbb {R}^{2n}} u^T\left\{ [\nabla _x V\cdot \nabla _v-v\cdot \nabla _x+\nu v\cdot \nabla _v-\sigma \Delta _v]P\right\} u f_{\infty }dxdv\nonumber \\&=\frac{2t\sigma }{\nu }\int _{\mathbb {R}^{2n}}\left\{ \sum _{i=1}^n (\partial _{v_i}u_{2})^T \frac{\partial ^2(\partial _{x_i}V)}{\partial x^2} u_{2} \right\} f_{\infty }dxdv. \end{aligned}$$

For the second term of (102) we compute

105 $$\begin{aligned}&-\int _{\mathbb {R}^{2n}} u^T\left\{ QP+PQ^T-\partial _t P\right\} uf_{\infty }dxdv\nonumber \\&=-\int _{\mathbb {R}^{2n}} \begin{pmatrix} u_1\\ u_2 \end{pmatrix}^T \small {\begin{pmatrix} 0&{} (t-2\varepsilon ^3t^3)\left( \frac{\partial ^2V}{\partial x^2}+c I\right) \\ (t-2\varepsilon ^3t^3)\left( \frac{\partial ^2V}{\partial x^2}+c I\right) &{} (-1+2\nu t-2\varepsilon ^2 t^2)\left( \frac{\partial ^2V}{\partial x^2}+c I\right) \end{pmatrix}}\begin{pmatrix} u_1\\ u_2 \end{pmatrix}f_{\infty }dxdv \nonumber \\&-\int _{\mathbb {R}^{2n}} \begin{pmatrix} u_1\\ u_2 \end{pmatrix}^T \small {\begin{pmatrix} 2\varepsilon ^2t^2(1-3\varepsilon )I&{} [2c\varepsilon ^3t^3+\nu \varepsilon ^2 t^2+2(1-\varepsilon )\varepsilon t]I\\ [2c \varepsilon ^3t^3+\nu \varepsilon ^2 t^2+2(1-\varepsilon )\varepsilon t]I&{}[2c \varepsilon ^2t^2+4\varepsilon \nu t-2\varepsilon ]I \end{pmatrix}}\begin{pmatrix} u_1\\ u_2 \end{pmatrix}f_{\infty }dxdv. \end{aligned}$$

Using the estimates

$$\begin{aligned}&-(t-2\varepsilon ^3t^3)\int _{\mathbb {R}^{2n}}u_1^T\left( \frac{\partial ^2V}{\partial x^2}+cI\right) u_2f_{\infty }dxdv\\ {}&\le \varepsilon ^3 t^2 |1-2\varepsilon ^3t^2|\int _{\mathbb {R}^{2n}}|u_1|^2f_{\infty }dxdv +\frac{|1-2\varepsilon ^3t^2|}{4\varepsilon ^3}\int _{\mathbb {R}^{2n}}|u_2|^2 \left| \left| \frac{\partial ^2V}{\partial x^2}+c I\right| \right| ^2f_{\infty }dxdv \end{aligned}$$

and

$$\begin{aligned}&-(-1+2\nu t-2\varepsilon ^2 t^2)\int _{\mathbb {R}^{2n}}u_2^T\left( \frac{\partial ^2V}{\partial x^2}+c I\right) u_2f_{\infty }dxdv\\ {}&\le |1-2\nu t+2\varepsilon ^2 t^2| \int _{\mathbb {R}^{2n}}|u_2|^2 \left| \left| \frac{\partial ^2V}{\partial x^2}+c I\right| \right| f_{\infty }dxdv, \end{aligned}$$

we get

106 $$\begin{aligned}&-\int _{\mathbb {R}^{2n}} u^T\left\{ QP+PQ^T-\partial _t P\right\} uf_{\infty }dxdv \nonumber \\&\le \int _{\mathbb {R}^{2n}} |u_2|^2\left[ \frac{|1-2\varepsilon ^3t^2|}{2\varepsilon ^3}\left| \left| \frac{\partial ^2V}{\partial x^2}+c I\right| \right| ^2+|1-2\nu t+2\varepsilon ^2 t^2|\left| \left| \frac{\partial ^2V}{\partial x^2}+c I\right| \right| \right] f_{\infty }dxdv \nonumber \\&-\int _{\mathbb {R}^{2n}} \begin{pmatrix} u_1\\ u_2 \end{pmatrix}^T \small {\begin{pmatrix} 2\varepsilon ^2t^2(1-3\varepsilon -\varepsilon |1-2\varepsilon ^2t^2|)I&{} [2c\varepsilon ^3t^3+\nu \varepsilon ^2 t^2+2(1-\varepsilon )\varepsilon t]I\\ [2c \varepsilon ^3t^3+\nu \varepsilon ^2 t^2+2(1-\varepsilon )\varepsilon t]I&{}[2c \varepsilon ^2t^2+4\varepsilon \nu t-2\varepsilon ]I \end{pmatrix}}\begin{pmatrix} u_1\\ u_2 \end{pmatrix}f_{\infty }dxdv. \end{aligned}$$

We fix $\varepsilon =\varepsilon (t_0)>0$ so that the element in the upper left corner of the matrix in (106) is positive for $t>0;$ more precisely we require

107 $$\begin{aligned} 1-3\varepsilon -\varepsilon |1-2\varepsilon ^2t^2|>0 \, \, \, \, \, \text { for all }\, \, \, t \in [0,t_0]. \end{aligned}$$

Then, the matrix in the last line of (106) can be estimated as

$$\begin{aligned} \begin{pmatrix} 2\varepsilon ^2t^2(1-3\varepsilon -\varepsilon |1-2\varepsilon ^2t^2|)I&{}[2c\varepsilon ^3t^3+\nu \varepsilon ^2 t^2+2(1-\varepsilon )\varepsilon t]I\\ [2c\varepsilon ^3t^3+\nu \varepsilon ^2 t^2+2(1-\varepsilon )\varepsilon t]I&{}[2c \varepsilon ^2t^2+4\varepsilon \nu t-2\varepsilon ]I \end{pmatrix}\\ \ge \begin{pmatrix} 0&{} 0\\ 0&{}[2c \varepsilon ^2t^2+4\varepsilon \nu t-2\varepsilon ]I-\frac{[2c\varepsilon ^2t^2+\nu \varepsilon t+2(1-\varepsilon )]^2}{2(1-3\varepsilon -\varepsilon |1-2\varepsilon ^2t^2|)}I \end{pmatrix}. \end{aligned}$$

Using this matrix inequality, we obtain from (106):

108 $$\begin{aligned}&-\int _{\mathbb {R}^{2n}} u^T\left\{ QP+PQ^T-\partial _t P\right\} uf_{\infty }dxdv\nonumber \\&\le \int _{\mathbb {R}^{2n}} |u_2|^2\left[ \frac{|1-2\varepsilon ^3t^2|}{2\varepsilon ^3}\left| \left| \frac{\partial ^2V}{\partial x^2}+c I\right| \right| ^2+|1-2\nu t+2\varepsilon ^2 t^2|\left| \left| \frac{\partial ^2V}{\partial x^2}+c I\right| \right| \right. \nonumber \\&\left. -2c\varepsilon ^2t^2-4\varepsilon \nu t+2\varepsilon +\frac{[2c\varepsilon ^2t^2+\nu \varepsilon t+2(1-\varepsilon )]^2}{2(1-3\varepsilon -\varepsilon |1-2\varepsilon ^2t^2|)}\right] f_{\infty }dxdv. \end{aligned}$$

(102), (103), (104), and (108) show that

$$\begin{aligned}&\frac{d}{dt}\int _{\mathbb {R}^{2n}}u^TPuf_{\infty }dxdv \le -2t\sigma \int _{\mathbb {R}^{2n}}\left\{ \sum _{i=1}^n (\partial _{v_i}u_2)^T\left( \frac{\partial ^2V}{\partial x^2}+c I\right) \partial _{v_i}u_2\right\} f_{\infty }dxdv\\&\quad +\frac{2t\sigma }{\nu }\int _{\mathbb {R}^{2n}}\left\{ \sum _{i=1}^n (\partial _{v_i}u_{2})^T \frac{\partial ^2(\partial _{x_i}V)}{\partial x^2} u_{2} \right\} f_{\infty }dxdv\\&\quad +\int _{\mathbb {R}^{2n}} |u_2|^2\left[ \frac{|1-2\varepsilon ^3t^2|}{2\varepsilon ^3}\left| \left| \frac{\partial ^2V}{\partial x^2}+c I\right| \right| ^2+|1-2\nu t+2\varepsilon ^2 t^2|\left| \left| \frac{\partial ^2V}{\partial x^2}+c I\right| \right| \right. \\&\quad \left. -2c\varepsilon ^2t^2-4\varepsilon \nu t+2\varepsilon +\frac{[2c\varepsilon ^2t^2+\nu \varepsilon t+2(1-\varepsilon )]^2}{2(1-3\varepsilon -\varepsilon |1-2\varepsilon ^2t^2|)}\right] f_{\infty }dxdv. \end{aligned}$$

As the matrix (6) is positive semi-definite, we have

$$\begin{aligned}&-2t\sigma \int _{\mathbb {R}^{2n}}\left\{ \sum _{i=1}^n (\partial _{v_i}u_2)^T\left( \frac{\partial ^2V}{\partial x^2}+cI\right) \partial _{v_i}u_2\right\} f_{\infty }dxdv\\&+\frac{2t\sigma }{\nu }\int _{\mathbb {R}^{2n}}\left\{ \sum _{i=1}^n (\partial _{v_i}u_{2})^T \frac{\partial ^2(\partial _{x_i}V)}{\partial x^2} u_{2} \right\} f_{\infty }dxdv\\&\le \tau t\int _{\mathbb {R}^{2n}}u_2^T\left( \frac{\partial ^2V}{\partial x^2}+cI\right) u_2f_{\infty }dxdv \le \tau t\int _{\mathbb {R}^{2n}}|u_2|^2\left| \left| \frac{\partial ^2V}{\partial x^2}+c I\right| \right| f_{\infty }dxdv. \end{aligned}$$

Subsequently,

109 $$\begin{aligned}&\frac{d}{dt}\int _{\mathbb {R}^{2n}}u^TPuf_{\infty }dxdv \nonumber \\&\quad \le \int _{\mathbb {R}^{2n}} |u_2|^2\left[ \frac{|1-2\varepsilon ^3t^2|}{2\varepsilon ^3}\left| \left| \frac{\partial ^2V}{\partial x^2}+c I\right| \right| ^2+(|1-2\nu t+2\varepsilon ^2 t^2|+\tau t)\left| \left| \frac{\partial ^2V}{\partial x^2}+c I\right| \right| \right. \nonumber \\&\qquad \left. -2c\varepsilon ^2t^2-4\varepsilon \nu t+2\varepsilon +\frac{[2c\varepsilon ^2t^2+\nu \varepsilon t+2(1-\varepsilon )]^2}{2(1-3\varepsilon -\varepsilon |1-2\varepsilon ^2t^2|)} \right] f_{\infty }dxdv. \end{aligned}$$

#### Step 4, decay of the functional $\Psi$:

We estimate the time derivative of (98): Combining (91), (97), and (109) yield

110 $$\begin{aligned}&\frac{d}{dt}\Psi (t,f(t) ) \nonumber \\&\le - \int _{\mathbb {R}^{2n}} |u_2|^2\left[ \left( \sigma e^{-8\tau n t} \gamma _1-\frac{|1-2\varepsilon ^3t^2|}{2\varepsilon ^3} \right) \left| \left| \frac{\partial ^2V}{\partial x^2}+c I\right| \right| ^2\right. \nonumber \\&\quad \left. -(|1-2\nu t+2\varepsilon ^2 t^2|+\tau t)\left| \left| \frac{\partial ^2V}{\partial x^2}+c I\right| \right| \right. \nonumber \\&\left. +2\sigma \gamma _2 +2c\varepsilon ^2t^2+4\varepsilon \nu t-2\varepsilon -\frac{[2c\varepsilon ^2t^2+\nu \varepsilon t+2(1-\varepsilon )]^2}{2(1-3\varepsilon -\varepsilon |1-2\varepsilon ^2t^2|)}\right] f_{\infty }dxdv. \end{aligned}$$

We fix $\gamma _1>0$ and $\gamma _2>0$ such that

111 $$\begin{aligned}&\left( \sigma e^{-8\tau n t } \gamma _1-\frac{|1-2\varepsilon ^3t^2|}{2\varepsilon ^3} \right) \left| \left| \frac{\partial ^2V}{\partial x^2}+c I\right| \right| ^2-(|-1+2\nu t-2\varepsilon ^2 t^2|+\tau t)\left| \left| \frac{\partial ^2V}{\partial x^2}+c I\right| \right| \nonumber \\&+2\sigma \gamma _2 +2c\varepsilon ^2t^2+4\varepsilon \nu t-2\varepsilon -\frac{[2c\varepsilon ^2t^2+\nu \varepsilon t+2(1-\varepsilon )]^2}{2(1-3\varepsilon -\varepsilon |1-2\varepsilon ^2t^2|)}\ge 0 \end{aligned}$$

for all $x \in \mathbb {R}^n$ and $t\in [0,t_0].$ We recall that we have fixed $\varepsilon =\varepsilon (t_0)$ so that (107) holds, which makes the above denominator positive. The existence of such $\gamma _1>0$ and $\gamma _2>0$ can be proven by the following arguments: We can consider the left hand side of (111) as a quadratic polynomial of $\left| \left| \frac{\partial ^2V}{\partial x^2}+c I\right| \right| \in [0, \infty ).$ As time *t* varies in a bounded interval $[0,t_0],$ the terms containing *t* are bounded. Therefore, we can choose large values for $\gamma _1=\gamma _1(t_0)$ and $\gamma _2=\gamma _2(t_0)$ so that this quadratic polynomial is non-negative for all $t\in [0,t_0].$

Consequently, we obtain that

$$\begin{aligned} \frac{d}{dt}\Psi (t, f(t))\le 0. \end{aligned}$$

Hence $\Psi (t, f(t))$ is decreasing and

112 $$\begin{aligned} \Psi (t,f(t))\le \Psi (0,f_0) \, \, \, \, \, \text { for all } \,\, \, \, t\in [0,t_0]. \end{aligned}$$

(101) and (112) show that

113 $$\begin{aligned} \displaystyle \int _{\mathbb {R}^{2n}}|u_1|^2 f_{\infty } dx dv \le \frac{1}{ \varepsilon ^3t^3}\int _{\mathbb {R}^{2n}} \left( \frac{f_0}{f_{\infty }} -1\right) ^2 \left( \gamma _1 \left| \left| \frac{\partial ^2V}{\partial x^2}+c I\right| \right| ^2+\gamma _2\right) f_{\infty }dxdv, \end{aligned}$$

114 $$\begin{aligned} \int _{\mathbb {R}^{2n}} |u_2|^2 f_{\infty } dxdv\le \frac{1}{\varepsilon t}\int _{\mathbb {R}^{2n}} \left( \frac{f_0}{f_{\infty }} -1\right) ^2 \left( \gamma _1 \left| \left| \frac{\partial ^2V}{\partial x^2}+c I\right| \right| ^2+\gamma _2\right) f_{\infty }dxdv,\nonumber \\ \end{aligned}$$

and

115 $$\begin{aligned} \int _{\mathbb {R}^{2n}} u_2^T \left( \frac{\partial ^2V}{\partial x^2}{+}cI\right) u_2 f_{\infty } dxdv \le \frac{1}{t}\int _{\mathbb {R}^{2n}} \left( \frac{f_0}{f_{\infty }}{-}1 \right) ^2 \left( \gamma _1 \left| \left| \frac{\partial ^2V}{\partial x^2}{+}c I\right| \right| ^2{+}\gamma _2\right) f_{\infty }dxdv.\nonumber \\ \end{aligned}$$

It is clear that there is a positive constant *C* such that

116 $$\begin{aligned} \gamma _1 \left| \left| \frac{\partial ^2V}{\partial x^2}+c I\right| \right| ^2+\gamma _2\le C\left( \left| \left| \frac{\partial ^2V}{\partial x^2}\right| \right| ^2+1\right) . \end{aligned}$$

(113), a proper linear combination of (114) and (115), and (116) imply the claimed estimates (11), (12). $\square$

##### Proof of Corollary 2.8

Theorems 2.3 and 2.7 show that, for $t\ge t_0>0,$

117 $$\begin{aligned}&\int _{\mathbb {R}^{2n}}\left( \frac{f(t)}{f_{\infty }}-1\right) ^2 f_{\infty }dxdv +\int _{\mathbb {R}^{2n}}\left| \nabla _{x}\left( \frac{f(t)}{f_{\infty }}\right) \right| ^2 f_{\infty }dxdv\nonumber \\&\quad +\int _{\mathbb {R}^{2n}}\nabla ^T_{v}\left( \frac{f(t)}{f_{\infty }}\right) \left( \frac{\partial ^2 V}{\partial x^2} +(1-\alpha _0)I\right) \nabla _{v}\left( \frac{f(t)}{f_{\infty }}\right) f_{\infty } dxdv \nonumber \\&\le C e^{-2\lambda (t-t_0)} \left[ \int _{\mathbb {R}^{2n}}\left( \frac{f(t_0)}{f_{\infty }}-1\right) ^2 f_{\infty }dxdv+\int _{\mathbb {R}^{2n}}\left| \nabla _{x}\left( \frac{f(t_0)}{f_{\infty }}\right) \right| ^2 f_{\infty }dxdv\right. \nonumber \\&\quad +\left. \int _{\mathbb {R}^{2n}}\nabla ^T_{v}\left( \frac{f(t_0)}{f_{\infty }}\right) \left( \frac{\partial ^2 V}{\partial x^2}+ (1-\alpha _0) I\right) \nabla _{v}\left( \frac{f(t_0)}{f_{\infty }}\right) f_{\infty }dxdv\right] \end{aligned}$$

holds with the constant *C* and the rate $\lambda$ given in Theorem 2.3. Using (11) and (12) at $t=t_0,$ we get

118 $$\begin{aligned} \displaystyle \int _{\mathbb {R}^{2n}}\left| \nabla _x \left( \frac{f(t_0)}{f_{\infty }}\right) \right| ^2f_{\infty } dx dv \le \frac{C_1}{t_0^{3}} \int _{\mathbb {R}^{2n}} \left( \frac{f_0}{f_{\infty }} -1\right) ^2 \left( \left| \left| \frac{\partial ^2V}{\partial x^2}\right| \right| ^2+1\right) f_{\infty }dxdv\nonumber \\ \end{aligned}$$

and

119 $$\begin{aligned}&\displaystyle \int _{\mathbb {R}^{2n}}\nabla _v^T \left( \frac{f(t_0)}{f_{\infty }}\right) \left( \frac{\partial ^2V}{\partial x^2}+(1-\alpha _0)I\right) \nabla _v \left( \frac{f(t_0)}{f_{\infty }}\right) f_{\infty } dx dv\nonumber \\&\quad \le \frac{C_2}{t_0}\int _{\mathbb {R}^{2n}} \left( \frac{f_0}{f_{\infty }} -1\right) ^2 \left( \left| \left| \frac{\partial ^2V}{\partial x^2}\right| \right| ^2+1\right) f_{\infty }dxdv. \end{aligned}$$

Combining (117), (118), and (119), we obtain (13). $\square$

## Data Availability

Data will be made available on reasonable request.

## Appendix

### Proof that Assumption 2.2’ Implies Assumption 2.2

Assume Assumption 2.2’ is satisfied. Let $$(u_1, u_2,...,u_{n+1})^T$$ be any vector in $$ \mathbb {R}^{n(n+1)},$$ where $$u_i $$ is a vector in $$ \mathbb {R}^n$$ for all $$i\in \{1,...,n+1\}. $$ We compute the quadratic form of the matrix (6)

$$\begin{aligned} \begin{pmatrix} u_1\\ u_2\\ .\\ .\\ .\\ u_{n+1} \end{pmatrix}^T \begin{pmatrix} \nu \left( \frac{\partial ^2 V(x)}{\partial x^2}+cI\right) &{}0&{}...&{}0&{}-\frac{1}{2}\frac{\partial ^2(\partial _{x_1}V(x))}{\partial x^2} \\ 0&{}\nu \left( \frac{\partial ^2 V(x)}{\partial x^2}+cI\right) &{}...&{}0&{} -\frac{1}{2}\frac{\partial ^2(\partial _{x_2}V(x))}{\partial x^2}\\ ...&{}...&{}...&{}...&{}...\\ 0&{}0&{}...&{}\nu \left( \frac{\partial ^2 V(x)}{\partial x^2}+cI\right) &{}-\frac{1}{2}\frac{\partial ^2(\partial _{x_n}V(x))}{\partial x^2}\\ -\frac{1}{2}\frac{\partial ^2(\partial _{x_1}V(x))}{\partial x^2}&{}-\frac{1}{2}\frac{\partial ^2(\partial _{x_2}V(x))}{\partial x^2}&{}...&{}-\frac{1}{2}\frac{\partial ^2(\partial _{x_n}V(x))}{\partial x^2}&{}\frac{\tau \nu }{2\sigma }\left( \frac{\partial ^2 V(x)}{\partial x^2}+cI\right) \end{pmatrix}\begin{pmatrix} u_1\\ u_2\\ .\\ .\\ .\\ u_{n+1} \end{pmatrix} \\ =\sum _{i=1}^n\left\{ \nu u_i^T\left( \frac{\partial ^2 V(x)}{\partial x^2} +cI\right) u_i-u^T_{i}\frac{\partial ^2 (\partial _{x_i}V(x))}{\partial x^2}u_{n+1}\right\} +\frac{\tau \nu }{2\sigma }u_{n+1}^T\left( \frac{\partial ^2 V(x)}{\partial x^2}+cI\right) u_{n+1}. \end{aligned}$$

semi-definite, it is enough to show the quadratic form above is non-negative. Assumption 2.2’ implies

$$\begin{aligned} \left| u^T_{i}\frac{\partial ^2 (\partial _{x_i}V(x))}{\partial x^2}u_{n+1}\right|{} & {} \le |u_{i}||u_{n+1}|\sqrt{\frac{2\tau \nu ^2}{n\sigma }}(\alpha (x)+ c)\le \nu (\alpha (x)+ c) |u_i|^2\\{} & {} +{\frac{ \tau \nu }{2n\sigma }}(\alpha (x)+ c)|u_{n+1}|^2. \end{aligned}$$

therefore, we get the desired result

$$\begin{aligned} \sum _{i=1}^n\left\{ \nu u_i^T\left( \frac{\partial ^2 V(x)}{\partial x^2}+cI\right) u_i-u^T_{i}\frac{\partial ^2 (\partial _{x_i}V(x))}{\partial x^2}u_{n+1} +\frac{\tau \nu }{2n\sigma }u_{n+1}^T\left( \frac{\partial ^2 V(x)}{\partial x^2}+cI\right) u_{n+1}\right\} \\ \ge \sum _{i=1}^n\left\{ \nu u_i^T\left( \frac{\partial ^2 V(x)}{\partial x^2}-\alpha (x)I\right) u_i+\frac{\tau \nu }{2n\sigma }u_{n+1}^T\left( \frac{\partial ^2 V(x)}{\partial x^2}-\alpha (x) I\right) u_{n+1}\right\} \ge 0. \quad \square \end{aligned}$$

### Matrix Inequalities for Section 5.1

#### Lemma 6.1

Let $\alpha _0>-\infty$ be the constant defined by (8), $a\in \mathbb {R}$ be some constant such that $a+\alpha _0>\frac{\nu ^2}{4},$ and $$P{:}{=}\begin{pmatrix} 2I&{}\nu I\\ \nu I&{} 2\frac{\partial ^2 V}{\partial x^2}+2a I \end{pmatrix}.$$ Then

120 $$\begin{aligned} c_1 P\le \begin{pmatrix} I&{}0\\ 0&{} \frac{\partial ^2 V}{\partial x^2}+(1-\alpha _0)I \end{pmatrix}\le c_2 P \end{aligned}$$

holds with $$c_1 {:}{=}\frac{1}{a+\alpha _0+1+\sqrt{(a+\alpha _0-1)^2+\nu ^2}}>0, \, \, \, \, \, c_2 {:}{=}\frac{a+\alpha _0+1+\sqrt{(a+\alpha _0-1)^2+\nu ^2}}{4(a+\alpha _0)-\nu ^2}>0.$$

#### Proof

We consider, for some $k\in \mathbb {R}$ to be chosen later as $\frac{1}{2c_{1,2}},$

$$\begin{aligned} A{:}{=}P-2k\begin{pmatrix} I&{}0\\ 0&{} \frac{\partial ^2 V}{\partial x^2}+(1-\alpha _0)I \end{pmatrix}=\begin{pmatrix} 2(1-k)I&{}\nu I\\ \nu I&{} 2(1-k)\left( \frac{\partial ^2 V}{\partial x^2}+(1-\alpha _0)I\right) +2(a+\alpha _0-1)I \end{pmatrix}. \end{aligned}$$

We check the (real) eigenvalues $\eta$ of the symmetric matrix *A* (depending on *k*). It is easy to check that $\eta = 2(1-k)$ is not an eigenvalue of *A*. If $\eta \ne 2(1-k),$ then we have the condition

$$\begin{aligned}&\det (A-\eta I)=\begin{vmatrix} 2(1-k)I-\eta I&\nu I \\\nu I&2(1-k)\left( \frac{\partial ^2 V}{\partial x^2}+(1-\alpha _0)I\right) +2(a+\alpha _0-1)I-\eta I \end{vmatrix}\\&\quad =\frac{1}{(2(1-k)-\eta )^n}\begin{vmatrix} 2(1-k)I-\eta I&0\\ \nu I&(2(1-k)-\eta )\left[ 2(1-k)\left( \frac{\partial ^2 V}{\partial x^2}+(1-\alpha _0)I\right) +2(a+\alpha _0-1)I-\eta I\right] -\nu ^2 I \end{vmatrix}\\&\quad =\det \left( (2(1-k)-\eta )\left[ 2(1-k)\left( \frac{\partial ^2 V}{\partial x^2}+(1-\alpha _0)I\right) +2(a+\alpha _0-1)I-\eta I\right] -\nu ^2 I\right) =0. \end{aligned}$$

If $\alpha _i,$ $i\in \{1,...,n\}$ are the eigenvalues of $\frac{\partial ^2 V}{\partial x^2},$ then the eigenvalues $\eta$ of *A* satisfy

121 $$\begin{aligned}{} & {} \prod _{i=1}^{n} \left( \eta ^2-2\eta [(1-k)(\alpha _i-\alpha _0+2)+a+\alpha _0-1]\right. \nonumber \\{} & {} \left. +4(1-k)^2(\alpha _i-\alpha _0+1)+4(1-k)(a+\alpha _0-1)-\nu ^2 \right) =0. \end{aligned}$$

**Right inequality of** (120): From (121), we see that *A* is positive semi-definite (i.e., all $\eta \ge 0$) if the following three conditions hold:

122 $$\begin{aligned} 1-k\ge 0, \, \, \, \, \, \, \text { (due to the first minor of} A) \end{aligned}$$

123 $$\begin{aligned} (1-k)(\alpha _i-\alpha _0+2)+a+\alpha _0-1\ge 0, \, \, \, \forall i\in \{1,...,n\}, \end{aligned}$$

124 $$\begin{aligned} 4(1-k)^2(\alpha _i-\alpha _0+1)+4(1-k)(a+\alpha _0-1)-\nu ^2 \ge 0, \, \, \, \, \forall i\in \{1,...,n\}. \end{aligned}$$

We set

$$\begin{aligned} k{:}{=}\frac{1}{2c_2}>0. \end{aligned}$$

Then, (122) holds:

125 $$\begin{aligned} 1-k=\frac{\sqrt{(a+\alpha _0-1)^2+\nu ^2}-(a+\alpha _0-1)}{2}>0. \end{aligned}$$

Using $\alpha _i\ge \alpha _0$ for all $i\in \{1,...,n\}$ we see that (123) also holds:

$$\begin{aligned} (1-k)(\alpha _i-\alpha _0+2)+a+\alpha _0-1\ge 2(1-k)+a+\alpha _0-1=\sqrt{(a+\alpha _0-1)^2+\nu ^2}>0. \end{aligned}$$

To verify (124) we estimate using $\alpha _i\ge \alpha _0$ for all $i\in \{1,...,n\}$ and (125)

$$\begin{aligned}&4(1-k)^2(\alpha _i-\alpha _0+1)+4(1-k)(a+\alpha _0-1)-\nu ^2\\&\ge 4(1-k)^2+4(1-k)(a+\alpha _0-1)-\nu ^2=0. \end{aligned}$$

Therefore, for *k* defined in (125), *A* is positive semi-definite. Hence, the inequality on the right hand side of (120) holds.

**Left inequality of** (120): Similarly, *A* is negative semi-definite if the following three conditions hold:

126 $$\begin{aligned} 1-k\le 0, \end{aligned}$$

127 $$\begin{aligned} (1-k)(\alpha _i-\alpha _0+2)+a+\alpha _0-1\le 0, \, \, \, \forall i\in \{1,...,n\}, \end{aligned}$$

128 $$\begin{aligned} 4(1-k)^2(\alpha _i-\alpha _0+1)+4(1-k)(a+\alpha _0-1)-\nu ^2 \ge 0, \, \, \, \, \forall i\in \{1,...,n\}. \end{aligned}$$

Setting

$$\begin{aligned} k{:}{=}\frac{1}{2c_1}>0 \end{aligned}$$

we find

129 $$\begin{aligned} 1-k=\frac{-\sqrt{(a+\alpha _0-1)^2+\nu ^2}-(a+\alpha _0-1)}{2}<0 \end{aligned}$$

and

$$\begin{aligned}{} & {} (1-k)(\alpha _i-\alpha _0+2)+a+\alpha _0-1\le 2(1-k)+a+\alpha _0-1\\{} & {} =-\sqrt{(a+\alpha _0-1)^2+\nu ^2}<0. \end{aligned}$$

Finally, we check using $\alpha _i\ge \alpha _0$ for all $i\in \{1,...,n\}$ and (129)

$$\begin{aligned}{} & {} 4(1-k)^2(\alpha _i-\alpha _0+1)\nonumber \\{} & {} +4(1-k)(a+\alpha _0-1)-\nu ^2 \ge 4(1-k)^2+4(1-k)(a+\alpha _0-1)-\nu ^2=0. \end{aligned}$$

Therefore, for *k* defined in (129), *A* is negative semi-definite. Hence, the inequality on the left hand side of (120) holds. $\square$

#### Remark 6.2

Lemma 6.1 proves the following matrix inequalities from Sect. 5.1:

- If $a=0$ and $\alpha _0>\frac{\nu ^2}{4},$ then (120) is the matrix inequality (56).
- If $a=\frac{\varepsilon ^2}{2}$ and $\alpha _0=\frac{\nu ^2}{4},$ then (120) is the matrix inequality (64).
- (120) coincides with the matrix inequality (75).

### Proof of Inequality (80)

We recall the assumption $\alpha _0< \frac{\nu ^2}{4}.$ We first rewrite

$$\begin{aligned}{} & {} \displaystyle A_2^{-1}=\frac{2\nu \min \{1, \alpha _0\}}{1+ \frac{\nu ^2}{2}-\alpha _0+ \sqrt{( \frac{\nu ^2}{2}-\alpha _0-1)^2+\nu ^2}}\\{} & {} =\frac{4 \min \{1, \alpha _0\}}{{\nu }+2(1-\alpha _0)\nu ^{-1}+ \sqrt{(\nu ^2-4\alpha _0)+4(\alpha _0+1)^2\nu ^{-2}}}, \\{} & {} \nu -\sqrt{\nu ^2-4 \alpha _0}=\frac{4\alpha _0}{\nu +\sqrt{\nu ^2-4\alpha _0}}. \end{aligned}$$

Then (80) is equivalent to

130 $$\begin{aligned} \frac{\alpha _0}{\nu +\sqrt{\nu ^2-4\alpha _0}}\ge \frac{\min \{1, \alpha _0\}}{\nu +2(1-\alpha _0)\nu ^{-1}+ \sqrt{(\nu ^2-4\alpha _0)+4(\alpha _0+1)^2\nu ^{-2}}}. \end{aligned}$$

If $\min \{1, \alpha _0\}=\alpha _0,$ then (130) is true because of

$$\begin{aligned} \nu +2(1-\alpha _0)\nu ^{-1}+ \sqrt{(\nu ^2-4\alpha _0)+4(\alpha _0+1)^2\nu ^{-2}}>\nu +\sqrt{\nu ^2-4\alpha _0}. \end{aligned}$$

If $\min \{1, \alpha _0\}=1,$ then (130) is equivalent to

$$\begin{aligned} \alpha _0 \nu -2\alpha _0(\alpha _0-1)\nu ^{-1}+ \alpha _0\sqrt{(\nu ^2-4\alpha _0)+4(\alpha _0+1)^2\nu ^{-2}}\ge \nu +\sqrt{\nu ^2-4\alpha _0}, \end{aligned}$$

or equivalently

$$\begin{aligned} (\alpha _0-1) (\nu ^2-2\alpha _0)\nu ^{-1}+ \alpha _0\sqrt{(\nu ^2-4\alpha _0)+4(\alpha _0+1)^2\nu ^{-2}}\ge \sqrt{\nu ^2-4\alpha _0}. \end{aligned}$$

The last inequality holds since

$$\begin{aligned} (\alpha _0-1) (\nu ^2-2\alpha _0)\nu ^{-1}\ge (\alpha _0-1) (\nu ^2-4\alpha _0)\nu ^{-1}\ge 0 \end{aligned}$$

and

$$\begin{aligned} \alpha _0\sqrt{(\nu ^2-4\alpha _0) +4(\alpha _0+1)^2\nu ^{-2}}>\sqrt{\nu ^2-4\alpha _0}. \end{aligned}$$

These two cases show that inequality (80) holds. $\square$

## Footnotes

1 For two matrices *A* and $B\in \mathbb {R}^{n \times n},$ $A\ge B$ means that $A-B$ is positive semi-definite.

2 For functions $\varphi =\varphi (t)$ and $\phi =\phi (t),$ $\varphi \asymp \phi$ as $t\rightarrow \infty$ means $\varphi =\mathcal {O}(\phi )$ and $\phi =\mathcal {O}(\varphi )$ as $t\rightarrow \infty .$

3 An eigenvalue is *defective* if its geometric multiplicity is strictly less than its algebraic multiplicity.
